# Remote Spatiotemporal Control of a Magnetic and Electroconductive Hydrogel Network via Magnetic Fields for Soft Electronic Applications

**DOI:** 10.1021/acsami.1c12458

**Published:** 2021-09-01

**Authors:** Anna Puiggalí-Jou, Ismael Babeli, Joan Josep Roa, Justin O. Zoppe, Jaume Garcia-Amorós, Maria-Pau Ginebra, Carlos Alemán, Jose García-Torres

**Affiliations:** † Departament d’Enginyeria Química, EEBE, Universitat Politècnica de Catalunya, C/ Eduard Maristany, 10-14, 08019 Barcelona, Spain; ‡ CIEFMA (Center for Research in Structural Integrity, Reliability and Micromechanics of Materials)-Department of Materials Science and Engineering, EEBE, Universitat Politècnica de Catalunya-BarcelonaTech, 08019 Barcelona, Spain; § Barcelona Research Center in Multiscale Science and Engineering, Universitat Politècnica de Catalunya, 08930 Barcelona, Spain; ∥ Department of Materials Science and Engineering, Universitat Politècnica de Catalunya (UPC), 08019 Barcelona, Spain; ⊥ Grup de Materials Orgànics, Departament de Química Inorgànica i Orgànica (Secció de Química Orgànica), Universitat de Barcelona, Martí i Franquès, 1, 08028 Barcelona, Spain; # Institut de Nanociència i Nanotecnologia (IN2UB), Universitat de Barcelona, 08028 Barcelona, Spain; ∇ Biomaterials, Biomechanics and Tissue Engineering Group, Department of Materials Science and Engineering, Universitat Politècnica de Catalunya (UPC), 08019 Barcelona, Spain; ○ Institute for Bioengineering of Catalonia (IBEC), Barcelona Institute of Science and Technology (BIST), Baldiri Reixac 10-12, 08028 Barcelona, Spain

**Keywords:** conductive hydrogel, magnetite nanoparticle, spatiotemporal control, magnetic field, soft electronics

## Abstract

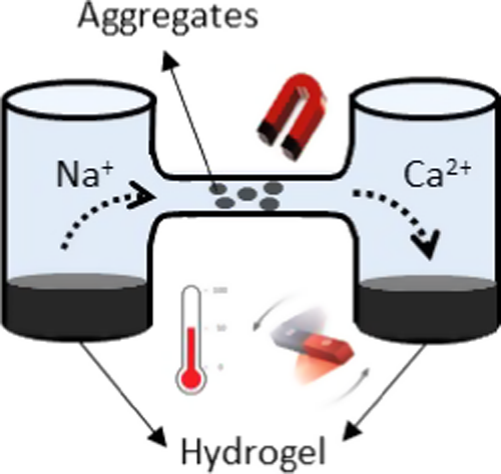

Multifunctional hydrogels are a class of materials offering new opportunities for interfacing living organisms with machines due to their mechanical compliance, biocompatibility, and capacity to be triggered by external stimuli. Here, we report a dual magnetic- and electric-stimuli-responsive hydrogel with the capacity to be disassembled and reassembled up to three times through reversible cross-links. This allows its use as an electronic device (e.g., temperature sensor) in the cross-linked state and spatiotemporal control through narrow channels in the disassembled state via the application of magnetic fields, followed by reassembly. The hydrogel consists of an interpenetrated polymer network of alginate (Alg) and poly(3,4-ethylenedioxythiophene) (PEDOT), which imparts mechanical and electrical properties, respectively. In addition, the incorporation of magnetite nanoparticles (Fe_3_O_4_ NPs) endows the hydrogel with magnetic properties. After structural, (electro)chemical, and physical characterization, we successfully performed dynamic and continuous transport of the hydrogel through disassembly, transporting the polymer–Fe_3_O_4_ NP aggregates toward a target using magnetic fields and its final reassembly to recover the multifunctional hydrogel in the cross-linked state. We also successfully tested the PEDOT/Alg/Fe_3_O_4_ NP hydrogel for temperature sensing and magnetic hyperthermia after various disassembly/re-cross-linking cycles. The present methodology can pave the way to a new generation of soft electronic devices with the capacity to be remotely transported.

## Introduction

Hydrogels, defined as three-dimensional (3D) polymeric networks infiltrated with water, are novel soft materials that have enabled great advances in diverse modern technologies including tissue engineering, drug delivery, biointegrated electronics, and soft robotics.^1−3^ Such advances have been made possible, in part, thanks to the similarities in physicochemical properties of hydrogels and biological tissues.^4,5^ While the softness and flexibility allow minimizing the mechanical mismatch between hydrogels and tissues, the high water content endows them with a wet and iron-rich environment, which is also characteristic of biological systems. In addition, hydrogels show remarkable biological properties (e.g., self-healing, self-adhesive, antimicrobial activity, biocompatibility, and biodegradability) as well as tunable mechanical (e.g., flexible, stretchable, etc.) and physical properties, making them a unique bridging material for the biological domain.^6,7^

Hydrogels are promising components of responsive hybrid materials, which react to chemical (e.g., ions and biomolecules) or physical (e.g., temperature, light, forces, and electric or magnetic fields) stimuli.^8−15^ For example, hydrogels with electrical conductivity are emerging as outstanding candidates for a new generation of soft electronics.^10,11^ Hydrogels are foreseen to close the gap between electronics (rigid, brittle, bulky) and biological systems (soft, flexible, tough) allowing seamless interfacing between them. To date, some wearable, implantable and bioresorbable hydrogel-based electronic devices have been successfully developed for physical (e.g., heart rate, temperature, and humidity) and chemical (e.g., glucose, lactate, pH, Na^+^, and K^+^) biomarkers monitoring, drug delivery, or electrostimulation of cells for tissue regeneration.^16−20^ Conductivity can be presented by either increasing ionic strength or the addition of electroconductive materials, such as nanoparticles (NPs),^21^ nanorods,^22^ nanowires,^23^ carbon nanotubes,^24^ or conducting polymers (CPs).^25^ Among these materials, CPs have recently garnered much attention to fabricate electroactive hydrogels due to their excellent electrical performance, extraordinary flexibility, and compatibility with other polymeric systems.^26,27^ In particular, poly(3,4-ethylenedioxythiophene) (PEDOT) is an ideal candidate not only because of its high ionic and electrical conductivity, but also its environmental and chemical stability (even in biological media), water dispersibility, biocompatibility, and electrochemical properties.^28−30^ A few examples of PEDOT hydrogels can be found in the literature for such applications as pressure sensors, drug delivery systems, biological studies, or tissue engineering.^31,32^

On the other hand, magnetic hydrogels have also recently received great attention to obtain functional structures with controllability, actuation, and spatiotemporal response regulated by an external magnetic field.^13,33^ These magnetoresponsive hydrogels have been used primarily in biomedical applications showing enhanced efficacy (e.g., magnetically controlled drug delivery, enhanced cell growth, and differentiation for tissue regeneration, magnetic actuation, or magnetic hyperthermia).^13,33^ Among the nanomaterials employed to afford magnetic properties, such as Co, Fe, NdFeB, and CoFe_2_O_4_,^34,35^ magnetite (Fe_3_O_4_) is most often employed for biomedical applications due to its attractive magnetic properties, biocompatibility, and noncytotoxicity.^36^

Based on the excellent performance of the single-stimulus-responsive systems, recent attention has been focused on multi-stimuli-responsive hydrogels to widen their application prospects. The most often investigated multifunctional hydrogels usually respond to two (or more) of the following stimuli: heat, NIR-light, pH, and/or electric fields.^37^ Less efforts have been dedicated to the fabrication of hydrogels simultaneously responding to magnetic and electric fields. For example, Liu et al. developed a magnetic-conductive bifunctional hydrogel by incorporating polydopamine (PDA)-chelated carbon nanotube–Fe_3_O_4_ nanohybrids within a polyacrylamide gel. The magnetic field was mainly used to align the nanohybrids in the field direction to obtain anisotropic hydrogels with a hierarchical structure analogous to biological tissues. The authors observed that the application of an external electric field-induced myoblast growth in the direction of nanohybrid orientation. Moreover, they proved the attraction of the hydrogel to a permanent magnet.^38^ Li and co-workers synthesized PEDOT/Fe_3_O_4_/polylactic acid-*co*-glycolic acid (PLGA) scaffolds by the in situ polymerization of EDOT monomers on the as-prepared electrospun Fe_3_O_4_/PLGA fibers. The hydrogel scaffold showed good electrical conductivity and superparamagnetic behavior, which allowed the growth of pre-osteoblasts with high cell viability through electric and magnetic stimulation.^39^ More recently, Wang et al. incorporated polypyrrole (PPy) and Fe_3_O_4_ NPs into a poly(vinyl alcohol) hydrogel showing favorable mechanical and electrical properties to be used as a strain sensor. Moreover, the magnetic properties of the hydrogel allowed its locomotion within a plastic tube to demonstrate its ability to be navigated across macroscopic length scales.^40^

Herein, we report a novel magneto- and electroresponsive, biocompatible hydrogel showing disassembly/re-cross-linking ability, which allows (1) its use as an electronic device (temperature sensor, magnetic hyperthermia) in the assembled macroscopic state, (2) transporting it in the disassembled state via magnetic fields as the hydrogel is broken apart into small aggregates, and (3) recovering the hydrogel functionality once re-cross-linked. It is important to highlight that the disassembly of the hydrogel will facilitate its motion through narrow channels (e.g., blood vessels, ureter), in which the cross-linked hydrogel could not pass; a unique characteristic not yet explored in previously reported hydrogels containing permanent covalent cross-links.^39,40^ The hydrogel is based on the interpenetration of alginate (Alg) and poly(3,4-ethylenedioxythiophene) (PEDOT) with magnetite nanoparticles (Fe_3_O_4_ NPs) embedded within the polymeric network. Each material plays a key role within the hydrogel. Alg is an ideal polysaccharide for hydrogel formation due to facile reversible cross-linking by divalent cations, biocompatibility, and biodegradability as well as water-solubility and processability. PEDOT is a water-dispersible conducting polymer that can provide electrical conductivity to hydrogels, which makes it compatible with biological applications. Fe_3_O_4_ NPs will impart soft magnetic properties to the hydrogel, allowing a fast response under the application of an external magnetic field, in addition to being biocompatible and nontoxic. Moreover, Fe_3_O_4_ NPs show other interesting properties (e.g., photocatalysis, contrast ability for use in magnetic resonance imaging (MRI), easy functionalization). In this work, we will explore the capacity of magnetite nanoparticles to generate heat (magnetic hyperthermia).

In this work, we have further successfully designed a strategy to spatially and temporally transport the hydrogel by external magnetic fields, while taking advantage of the disassembly/re-cross-linking ability of the hydrogel and its magnetic responsiveness. Thus, the macroscopic hydrogel can be easily disassembled by replacing Ca^2+^ with monovalent ions, such as Na^+^, leading to a suspension of aggregates composed of polymeric chains and Fe_3_O_4_ NPs. The polymer–Fe_3_O_4_ NP aggregates can be magnetically propelled to the target and, once there, the multifunctional hydrogel can be recovered by re-cross-linking the polymeric chains through the addition of Ca^2+^ ions. It is important to mention that this process can be performed up to 3 disassembly/re-cross-linking cycles. The likely explanations for this 3-cycle limit and potential solutions to increase the number of cycles will be elaborated on in the Results and Discussion section.

The realization of dual magnetically and electrically responsive hydrogels showing mechanical flexibility, biocompatibility, biodegradability, and injectability, as well as the capacity to be spatiotemporally controlled via magnetic fields, make them ideal candidates for a new generation of advanced multifunctional electronic materials and devices. The strategy developed for the remote control of soft devices represents a conceptually novel technological challenge, opening the door to an unprecedented number of applications from diagnostic and treatment of diseases to environmental remediation, energy devices, or soft robotics.

## Results and Discussion

### Conceptualization, Design, and Synthesis of the Dual-Responsive Hydrogel with Remote Spatiotemporal Control

**Figure 1:**
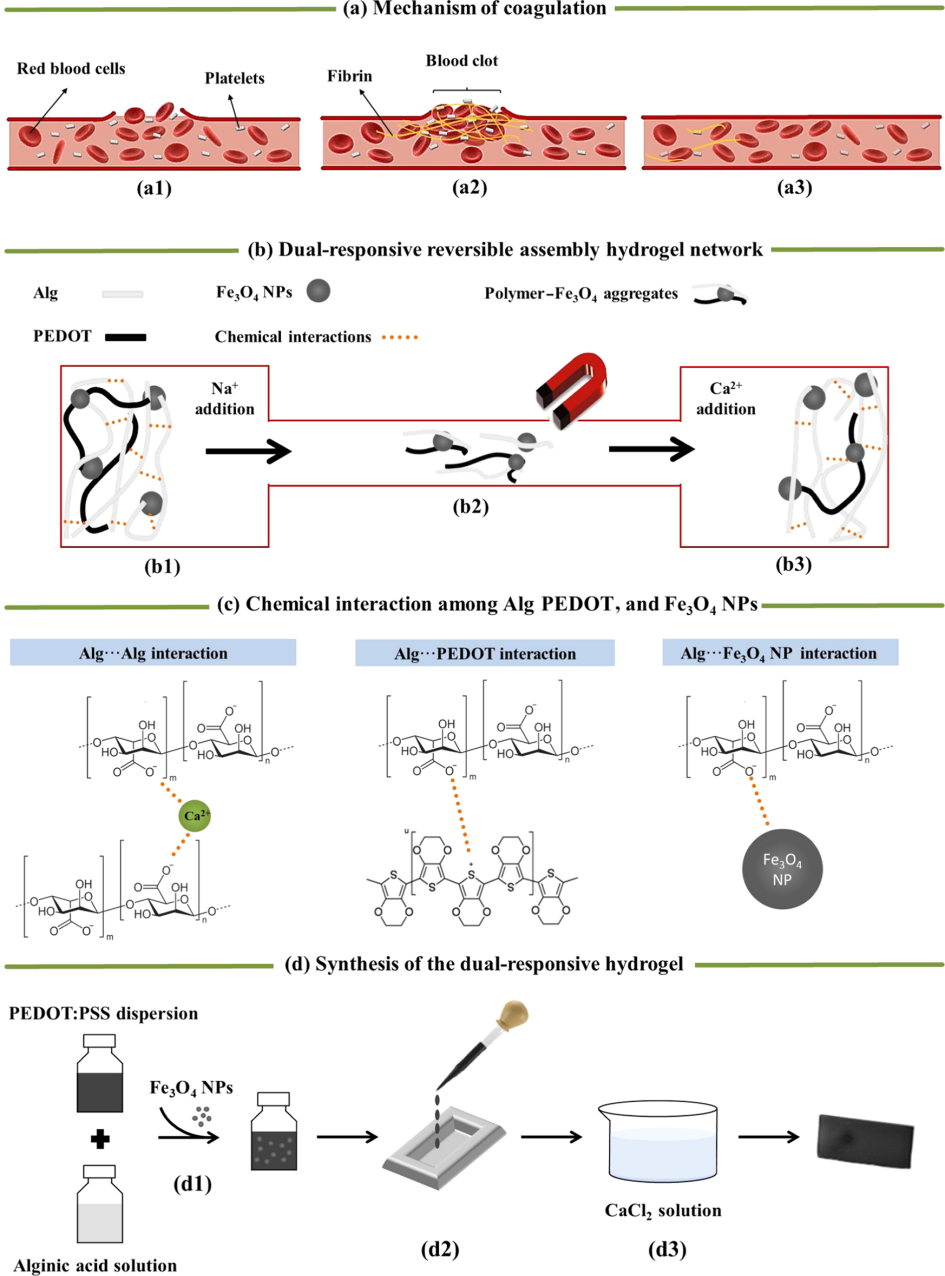
(a) Scheme showing the mechanism of coagulation: (a1) transport of the platelets and other blood cells (e.g., red blood cells) toward the damaged area, (a2) formation of the blood clot (platelets, red blood cells, fibrin-based mesh) and (a3) disassembly of the blood clot once the vessel is healed. (b) Scheme showing the strategy to transport the functional hydrogel through a narrow channel: (b1) disassembly with phosphate buffer saline (PBS) solution as Ca^2+^ ions are replaced by Na^+^ ions. The interactions between alginate chains are broken and the hydrogel disintegrates into the polymer–Fe_3_O_4_ NP aggregates. (b2) Magnetic transport of the aggregates through a narrow channel and (b3) re-cross-linking using CaCl_2_ solution. (c) Scheme showing the chemical interactions established between the different hydrogel components: Alg···Alg, Alg···PEDOT, and Alg···Fe_3_O_4_ NP electrostatic interactions. (d) Scheme illustrating the synthesis procedure of the dual magnetically and electrically responsive hydrogel: (d1) mixture of the Alg, PEDOT, and Fe_3_O_4_ NPs to obtain a homogeneous dispersion and (d2) pouring the mixture into a mold and (d3) cross-linking in a CaCl_2_ solution.

Nature has been a source of inspiration for scientists and engineers to tackle the great challenges of the future as it has developed efficient, sustainable materials and processes as a result of natural selection and competition.^41^ One such process is hemostasis, the mechanism utilized by our body to prevent and stop bleeding.^42^ Briefly, the mechanism of coagulation, schematically shown in Figure 1a, involves the motion, adhesion, and aggregation of platelets to the edges of the damaged tissue of the vessel, forming a plug. Then, the thrombin enzyme converts fibrinogen into fibrin, which further polymerizes into a gel mesh. This mesh embeds the aggregated platelets, as well as other blood cells (e.g., red blood cells), and confers mechanical stability to the plug, yielding the blood clot. Once the injured vessel is healed, the fibrin hydrogel is broken down by the plasmin enzyme, which disassembles the clot, a process called fibrinolysis. Thus, the hemostasis–fibrinolysis processes involve the assembly and disassembly of a composite hydrogel, as well as the motion of the different components either toward the damaged vessel (e.g., the target) during hemostasis or away from it during fibrinolysis.^42^

This biological process has inspired us to design a bifunctional magnetically and electrically responsive hydrogel with the ability to be disassembled, remotely transported via magnetic fields, and re-cross-linked at a new target position. Such dual functionality will allow the hydrogel and/or device in the assembled state to be disassembled into the polymer–Fe_3_O_4_ NP aggregates and magnetically steered through regions, in which the cross-linked hydrogel would otherwise not be able to pass. This process is also schematically illustrated in Figure 1b. The hydrogel consists of Fe_3_O_4_ NPs (analogous to the platelets, red blood cells) embedded in an interpenetrated network of PEDOT and Alg chains (resembling the fibrinogen mesh) that can be cross-linked/disassembled (analogous to the hemostasis–fibrinolysis process) (Figure 1c). Despite the similarities to the blood clot, the synthetic composite hydrogel and the strategy to remotely control its position using external electromagnetic fields could open the door to unprecedented applications. Nevertheless, one drawback of the current system is that the cross-linking/disassembly process can only be performed up to 3 cycles.

The hydrogel is formed by the electrostatic interaction between Alg and PEDOT chains (as shown by density functional theory (DFT) calculations ),^31^ followed by cross-linking of Alg with solutions of divalent cations, such as CaCl_2_. Magnetite nanoparticles are present during Alg cross-linking, such that they are embedded into the hydrogel. Fe_3_O_4_ NPs favor the entanglement with polymeric chains to form a PEDOT/Alg/Fe_3_O_4_ network not only due to physisorption but also because the Fe^2+^ and Fe^3+^ ions of magnetite electrostatically interact with the anionic carboxylate groups of Alg.^36^ While PEDOT renders the hydrogel electrically conductive, Alg allows reversible cross-linking and therefore the ability of the hydrogel to be assembled and disassembled. Moreover, the Fe_3_O_4_ NPs permit the wireless transport of the disassembled hydrogel aggregates toward the target using external magnetic fields. Such strategies could be useful for applications not only on the macroscale (e.g., soft electronics) but also on the micro- and nanoscale (e.g., micro/nanorobotics), as the magnetic transport of the components in the disassembled state has already proven successful at such small length scales.^43^

The overall procedure for the preparation of the electric and magnetic hydrogels is shown in Figure 1d. First, equal volumes of a poly(3,4-ethylenedioxythiophene):poly(styrene sulfonate) (PEDOT:PSS) dispersion (1.3 wt %) and an alginic acid solution (3.9 wt %) is vigorously mixed until achieving a homogenous distribution of both components (ca. 20 min stirring). After that, magnetite powder is added to the previous dispersion in different amounts (5, 10, or 20 wt % with respect to the solid content) and homogenized. During this step, Alg interacts electrostatically not only with PEDOT chains but also with the Fe^2+^/Fe^3+^ ions of magnetite nanoparticles. Finally, the resultant dispersion is poured into a homemade silicone mold, followed by immersion in a CaCl_2_ solution (3 wt %) to cross-link the Alg and form the PEDOT/Alg/Fe_3_O_4_ composite hydrogel. After gelling, the hydrogels are thoroughly washed with deionized water to remove the excess of Alg. For disassembly, the hydrogels are immersed in PBS for a few minutes to allow the diffusion of Na^+^ ions inside the hydrogel and the replacement of Ca^2+^ ions, breaking the hydrogel down into polymer–Fe_3_O_4_ NP aggregates. The replacement of Ca^2+^ ions with Na^+^ ions takes place due to the Na^+^ concentration gradient.

### Characterization of PEDOT/Alg/Fe_3_O_4_ Hydrogels

#### Morphological and Structural Characterization

**Figure 2:**
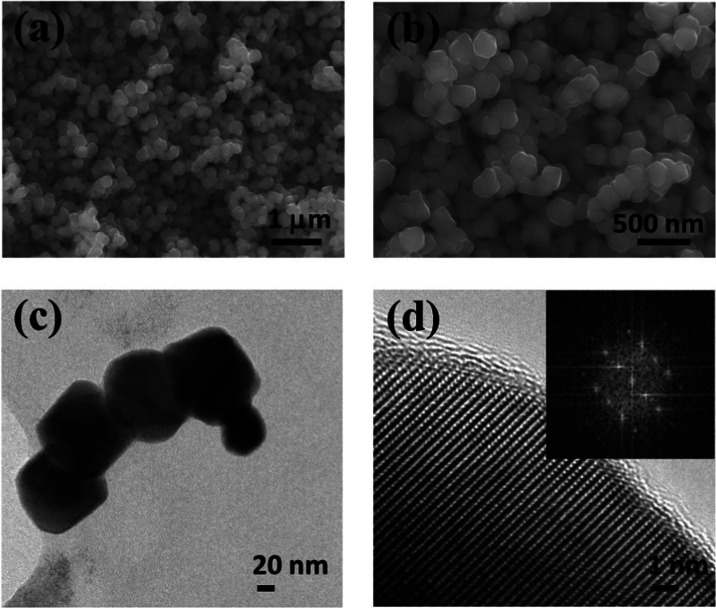
(a, b) SEM, (c) TEM, and (d) high-resolution TEM (HRTEM) images of Fe_3_O_4_ nanoparticles. Inset in (d) shows the fast Fourier transformation (FFT).

First, the morphology of Fe_3_O_4_ NPs in powder form was characterized via electron microscopy. Figure 2 shows scanning electron (SEM) and transmission electron micrographs (TEM) of the commercial magnetite nanoparticles employed. While low-magnification SEM micrographs showed homogeneous particles with diameters between 150 and 200 nm, higher-magnification SEM and TEM micrographs evidenced that the particles were not completely spherical but faceted. For example, cubic particles were even detected (Figure S1). Such morphologies are interesting, as faceted shapes can favor not only the entanglement of the polymeric chains and magnetite nanoparticles through the edges of different faces but also enhance the magnetic heating efficiency compared to spherical particles for magnetic hyperthermia applications.^44^ On the other hand, particles with diameters smaller than 50 nm were also detected, which were below the reported critical size of 76 nm for the transition from superparamagnetic (single magnetic domain) to ferromagnetic (multimagnetic domain) behavior.^45^

The analysis of the high-resolution transmission electron microscopy (HRTEM) images of the nanoparticles showed a lattice image with a fringe pattern (Figures 2d and S1). Moreover, the polycrystalline nature of the nanoparticles was observed as multiple directions of lattice fringes within one particle.^45^ An interplanar spacing (*d*) was directly measured from the high-resolution images (0.25 nm), which corresponded to the (311) plane of the face-centered cubic lattice with inverse spinel structure (spatial group *Fd*3̅). Fast Fourier transform (FFT) analyses were also performed on different regions of the HRTEM images (see the inset in Figure 2d). The diffraction spots were indexed using the reported lattice parameter data from the corresponding powder diffraction file (PDF# JCPDS 19-0629).^46^ Interplanar *d*-spacings of 0.1480, 0.1612, 0.2088, 0.2523, and 0.2959 nm matched perfectly with the (440), (511), (400), (311), and (111) planes of the inverse spinel cubic structure, respectively. These results are in agreement with those found in the literature.^45,47^

Magnetite was loaded into the hydrogel in different amounts (5, 10, or 20 wt %) to study its effect on the properties of the hydrogel and its potential applicability in the wireless transport of the disassembled polymeric network. To that end, the morphology and the distribution of the different components within the hydrogels were characterized by SEM and energy-dispersive X-ray spectroscopy (EDX), respectively. As a reference, PEDOT/Alg hydrogels in the absence of magnetite nanoparticles showed an open and interconnected porous structure (Figure S2) with an average pore size of 9 ± 2 μm, as estimated by SEM. On the other hand, EDX mapping analysis indicated that PEDOT and Alg chains were homogeneously distributed within the hydrogel, as the signals of characteristic elements for each polymer (sulfur for PEDOT and calcium for Alg) were equally detected and superimposed along the entire hydrogel cross section. Thus, PEDOT and Alg were well mixed and formed an interpenetrating polymeric network. Such homogeneity and network formation are also beneficial in obtaining homogenous and isotropic properties throughout the entire hydrogel.

**Figure 3:**
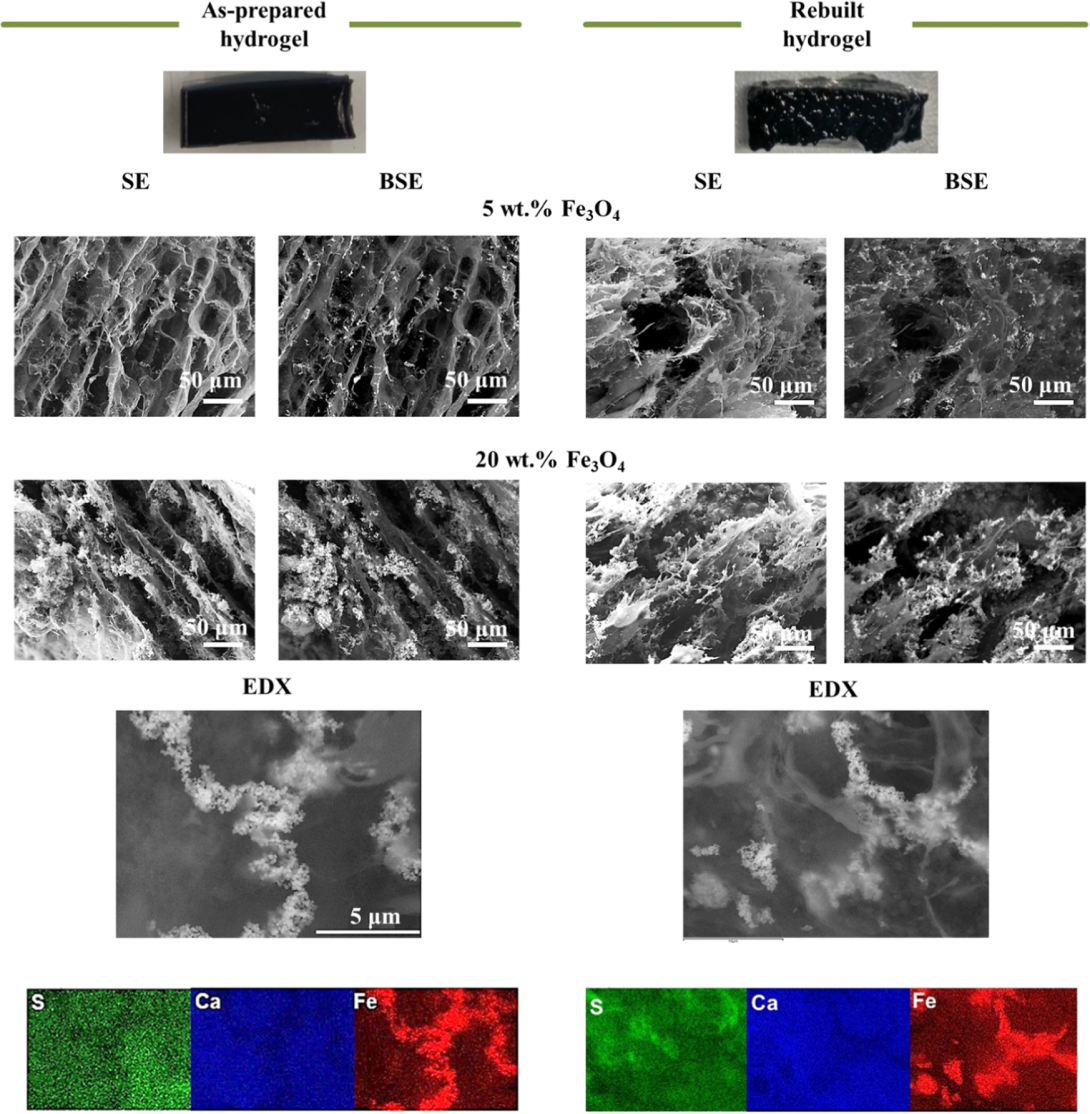
Photographs of the PEDOT/Alg/10 wt % Fe_3_O_4_ as-prepared and rebuilt hydrogels. Secondary electron (SE) and backscattered electron (BSE) micrographs for different magnetite contents in the hydrogels (5 and 20 wt % Fe_3_O_4_ NPs). The SEM image and the corresponding EDX mapping (elements: S, Ca, Fe) for the PEDOT/Alg/10 wt % Fe_3_O_4_ as-prepared and rebuilt hydrogels.

The incorporation of magnetite completely changed the PEDOT/Alg microstructure, in which a more closed structure with less pore interconnectivity was observed (Figure 3). This change was even more evident as the content of Fe_3_O_4_ NPs was increased in the hydrogel. In this case, a more entangled polymeric network was favored not only because of the presence of the nanoparticles themselves but also by their faceted morphology, providing more anchoring points and electrostatic interactions with Alg chains. It is also worth mentioning that the presence of the nanoparticles, at least up to 20 wt %, did not disrupt the continuity of the polymeric matrix, allowing a continuous and mechanically stable composite hydrogel. The presence of nanoparticles was detected using the backscattered electron (BSE) imaging mode, which showed a contrasted image between the low atomic mass elements (C, H, S) present in PEDOT and Alg (darker areas) and the high atomic mass of Fe in magnetite (brighter areas) (Figure 3). From the BSE images, we observed some aggregates due to the magnetic attraction between the nanoparticles; however, such aggregates were evenly distributed throughout the entire hydrogel. EDX mapping corroborated the presence of such Fe_3_O_4_ clusters, as a more intense Fe signal was observed. The EDX mapping also showed that the Fe signal was present throughout the entire hydrogel, suggesting a homogeneous distribution of the nonagglomerated particles. The interaction of the Fe_3_O_4_ NPs with the polymeric network can prevent but not completely avoid magnetic attraction and therefore aggregation of some of the particles. Moreover, the presence of the nanoparticles did not affect the homogeneous distribution of both PEDOT and Alg since the sulfur and calcium signals were detected throughout the hydrogel. Although some regions of lower intensity were observed in the S- and Ca-EDX mappings, they mainly corresponded to the areas in which the Fe_3_O_4_ NPs were aggregated; therefore, the S and Ca signals were partially masked by the Fe signal in those regions. On the other hand, it is important to clarify that calcium is a characteristic signal of Alg since Ca^2+^ ions only cross-link the alginate chains and not PEDOT. Moreover, since the excess of Ca^2+^ ions were removed by thoroughly washing the hydrogel after cross-linking, only the Ca^2+^ electrostatically bound to the −COO^–^ groups of Alg were present in the hydrogel.

From a macroscopic point of view, it was easy to manipulate and handle all of the hydrogels, showing mechanical integrity and flexibility. The hydrogel mechanical stability is mainly provided by the interaction between the different components of the hydrogel: (i) the cross-linking of Alg chains induced by the electrostatic interaction between divalent Ca^2+^ cations and the COO^–^ groups; (ii) the electrostatic interaction between the Alg and PEDOT chains; and (iii) the electrostatic interaction between Alg and magnetite. On the other hand, the presence of the magnetic nanoparticles themselves, favoring the entanglement with the polymeric chains, also contributes to the mechanical integrity of the hydrogels. In general, the macroscopic appearance of the different hydrogels was similar, except that the color of PEDOT/Alg hydrogels becomes slightly darker when they are loaded with magnetite nanoparticles. Moreover, all PEDOT/Alg/Fe_3_O_4_ samples responded to the presence of a FeNdB (iron–neodymium–boron) permanent magnet, indicating the successful incorporation and homogenous distribution of the magnetite nanoparticles, as previously evidenced by SEM and EDX.

After morphological analyses, the reversibility of the cross-linking and its impact on the morphology of the hydrogel was investigated. First, the hydrogel was disassembled by mixing with phosphate buffer saline (PBS) solution, leading to a set of PEDOT/Alg/Fe_3_O_4_ aggregates after a few minutes. Since most of the aggregates remained dispersed in solution, they were subsequently collected at the bottom of the vial with a permanent magnet to recover as much aggregates as possible (Figure S3). Finally, the PBS solution was replaced by a CaCl_2_ solution to rebuild the hydrogel. Figure 3 shows a photograph of the rebuilt hydrogel. From a macroscopic point of view, the rebuilt hydrogels had a more compact appearance than the as-prepared hydrogels. The SEM examination of the cross-sectional morphology revealed a more closed microstructure, showing lower porosity compared to the as-prepared hydrogels. We repeated the disassembly/re-cross-linking process up to three times and we did not observe any changes in the microstructure, except an increase in the proportion of magnetite NPs (Figure S4). This result suggested that some of the Alg and PEDOT polymeric chains were not recovered with the magnet during this process and remained dispersed in solution (Figure S3). We thus deduced that re-cross-linking the hydrogel a fourth time would lead to low mechanical integrity and, therefore, we concluded that the hydrogels can only withstand three disassembly/re-cross-linking cycles. The higher macroscopic and microscopic compactness observed in the re-cross-linked hydrogels was attributed not only to the presence of the magnet but also to the fact that the magnetic nanoparticles were more efficiently attracted by the magnet due to the decrease in the Alg and PEDOT content in the hydrogel after each cycle.

**Figure 4:**
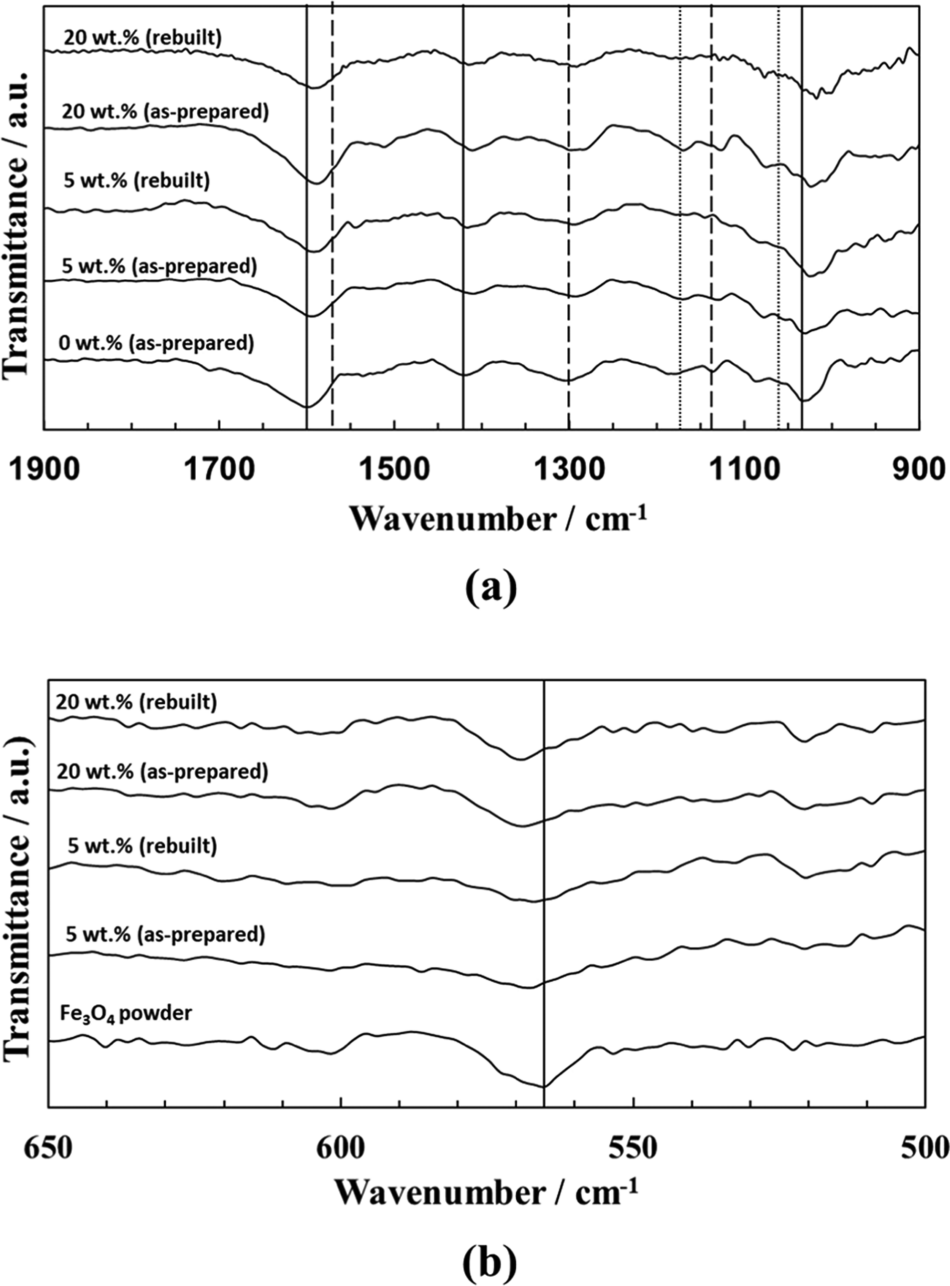
(a) ATR-FTIR spectra of the PEDOT/Alg/Fe_3_O_4_ hydrogels containing different amounts of magnetite before (as-prepared) and after (rebuilt) three disassembly/re-cross-linking cycles. The solid, dashed, and dotted lines correspond to Alg, PEDOT, and PSS, respectively. (b) ATR-FTIR spectra (region 650–500 cm^–1^) of the magnetite powder and the PEDOT/Alg/Fe_3_O_4_ hydrogels containing different amounts of magnetite (5 and 20 wt %) before (as-prepared) and after (rebuilt) three disassembly/re-cross-linking cycles. The solid line corresponds to Fe–O stretching of magnetite.

Attenuated total reflectance Fourier transform infrared spectroscopy (ATR-FTIR) investigations of the different hydrogels allowed the detection of the different components present in the feedstock solutions employed in their preparation (Figure 4). In the PEDOT/Alg hydrogel, we observed characteristic peaks of Alg at 1598, 1419, and 1028 cm^–1^, attributed to the asymmetric and symmetric stretching vibrations of COO and C–O, respectively. Meanwhile, PEDOT:PSS showed characteristic peaks at 1564 cm^–1^ for the C=C stretching of the thiophene ring, and 1300 and 1135 cm^–1^ for the C–C vibrations of the fused dioxane ring in PEDOT. Moreover, the peaks at 1162 and 1060 cm^–1^ were attributed to the S–O and *S*-phenyl vibrations of PSS chains, respectively (Figure 4a).^48^ On the other hand, the presence of Fe_3_O_4_ NPs was detected not only by the characteristic fingerprint of the Fe–O stretching at 565 cm^–1^ (Figure 4b) but also by the shift in the peak positions of the other hydrogel components. This shift was indicative of the interaction between the Fe_3_O_4_ and the polymeric chains as previously reported.^49^ The spectra were further recorded in different areas of the hydrogel, confirming the homogeneous distribution of both polymers and the nanoparticles. These results agreed with those previously shown by EDX analysis. In addition, we performed the ATR-FTIR analysis on the rebuilt hydrogels (Figure 4a,b). As expected, all of the spectra showed the characteristic peaks of both PEDOT and Alg and the presence of the magnetite nanoparticles at the same positions as those obtained for the as-prepared samples. The only detectable difference was a slight increase in the Fe_3_O_4_ peak intensity in the re-cross-linked hydrogels, which is in agreement with the observations of the EDX analyses.

#### Rheological and Mechanical Characterization

**Figure 5:**
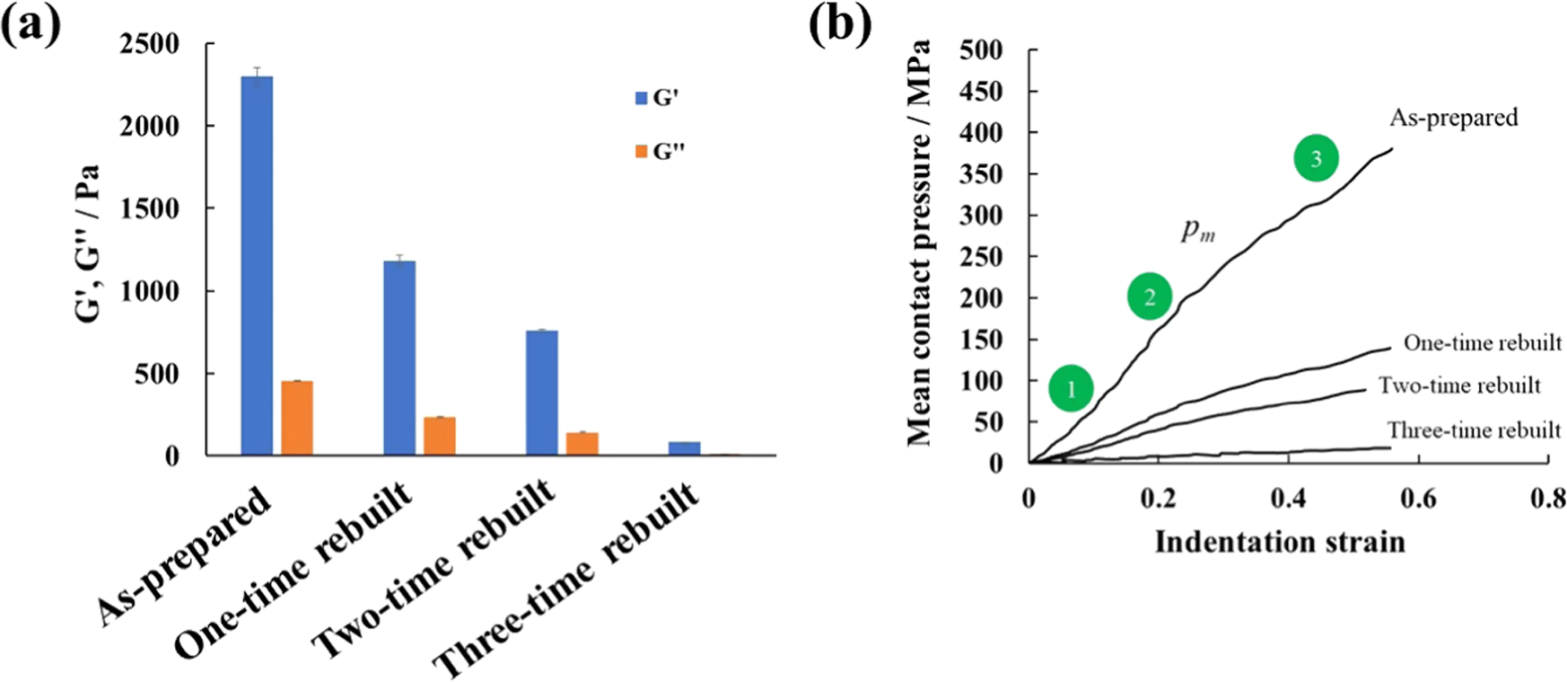
(a) Storage (*G*′) and loss moduli (*G*″) at room temperature and a frequency of 1 Hz and (b) stress–strain curves obtained via nanoindentation for the as-prepared, one-, two- and three-cycle rebuilt PEDOT/Alg/10 wt % Fe_3_O_4_ NP hydrogels.

The effect of the disassembly/re-cross-linking cycles on the bulk mechanical properties of the composite hydrogels was first assessed by rheological analysis. As observed in Figure 5a, the storage modulus (*G*′) was higher than the loss modulus (*G*″) in the as-prepared as well as the rebuilt hydrogels (containing 10 wt % Fe_3_O_4_ NPs), indicating their dominant behavior as viscoelastic solids. Moreover, both *G*′ and *G*″ decreased with the number of disassembly/re-cross-linking cycles, indicating that the stiffness of the composite hydrogels progressively decreased. This result was attributed to an apparent lower cross-linking density due to the loss of the Alg polymer after each cycle (as detected by EDX analysis) and, thus, a loss in the mechanical integrity of the hydrogels. Quantitatively, *G*′ decreased from 2301 Pa for the as-prepared hydrogel to 1181, 761, and 83 Pa for the one-, two- and three-cycle rebuilt hydrogels, respectively. Such a decrease implied a reduction in the storage modulus of 49, 67, and 96% with respect to the as-prepared hydrogel *G*′ value. The *G*′ and *G*″ values recorded over time were, nevertheless, stable and did not significantly change during the experiment duration (Figure S5).^50^

To better quantify the stiffness of the composite hydrogels, their mechanical properties were further analyzed by spherical nanoindentation. Figure 5b shows the indentation stress, also known as the mean contact pressure (*p*_m_), as a function of the indentation strain (*a*/*R*) for the as-prepared and one-, two- and three-cycle rebuilt PEDOT/Alg/10 wt % Fe_3_O_4_ hydrogels. The indentation strain (*a*/*R*) is defined as the ratio between the contact point (*a*) and the tip indenter radius (*R*). By constructing the indentation stress–strain curve (*p*_m_ vs *a*/*R*) and determining the *p*_m_ for a given indentation strain, other mechanical parameters (i.e., yield strength, σ_ys_, and maximum tensile and shear stress, σ_tm_ and τ_max_, respectively) were determined according to the following equations^51,52^
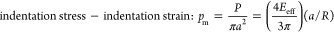
1
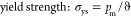
2
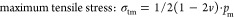
3
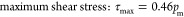
4where θ is the constraint factor and υ is the Poisson’s ratio. The constraint factor θ depends on the geometry of the indenter and friction at the interface; in this case equal to 1.1.

The *p*_m_–*a*/*R* curves exhibited serrated regions, which may be related to the presence of internal porosity heterogeneously distributed throughout the samples. Furthermore, all curves can be divided into three different regions, labeled from 1 to 3 in Figure 5b. At the start of the spherical indentation test (region 1), the indentation process was essentially only taking place on the rough and porous material surface, in which the indenter–hydrogel contact was not fully uniaxial. On the other hand, region 2 is related to the primary elastic behavior until the applied load reaches the yield stress of the material and, finally, region 3 corresponds to the elastoplastic deformation of the hydrogel.

The results, shown in Table 1, indicate that the mechanical properties of the hydrogels decreased with the number of disassembly/re-cross-linking cycles, beginning with a relatively stiff hydrogel and ending with a much softer material. For example, the elastic modulus (*E*) decreased from 681 MPa for the as-prepared hydrogel to 324, 225, and 34 MPa for the one-, two- and three-cycle rebuilt hydrogels, respectively. As noted above, the decrease in mechanical strength was most likely due to the loss of Alg after each subsequent cross-linking/disassembly cycle, which plays a critical role in maintaining a strong polymeric network within the composite hydrogels. These results agree with those determined by EDX analysis and rheology experiments, overall demonstrating the 3-cycle limitation of the hydrogels.

**Table 1:** Summary of Mechanical Properties (*E*, *p*_m_, σ_ys_, σ_tm_, and τ_max_) Obtained from the Spherical Indentation Tests for the As-Prepared, One-, Two-, and Three-Cycle Rebuilt PEDOT/Alg/10 wt % Fe_3_O_4_ NP Hydrogels

specimen	*E* (MPa)	*p*_m_ (MPa)	σ_ys_ (MPa)	σ_tm_ (MPa)	*τ*_max_ (MPa)
as-prepared hydrogel	681 ± 14	247.5 ± 5.0	227.7 ±4.6	47.0 ± 1.0	113.9 ± 2.3
one-cycle rebuilt hydrogel	324 ± 10	77.9 ± 1.8	70.8 ± 0.9	15.6 ± 0.3	43.6 ± 0.5
two-cycle rebuilt hydrogel	225 ± 8	42.7 ± 0.9	38.8 ± 0.7	8.54 ± 0.2	23.9 ± 0.7
three-cycle rebuilt hydrogel	34.3 ± 3	12.9 ± 1.3	11.9 ± 1.2	2.45 ± 0.2	5.93 ± 0.6

#### Electrochemical Characterization

**Figure 6:**
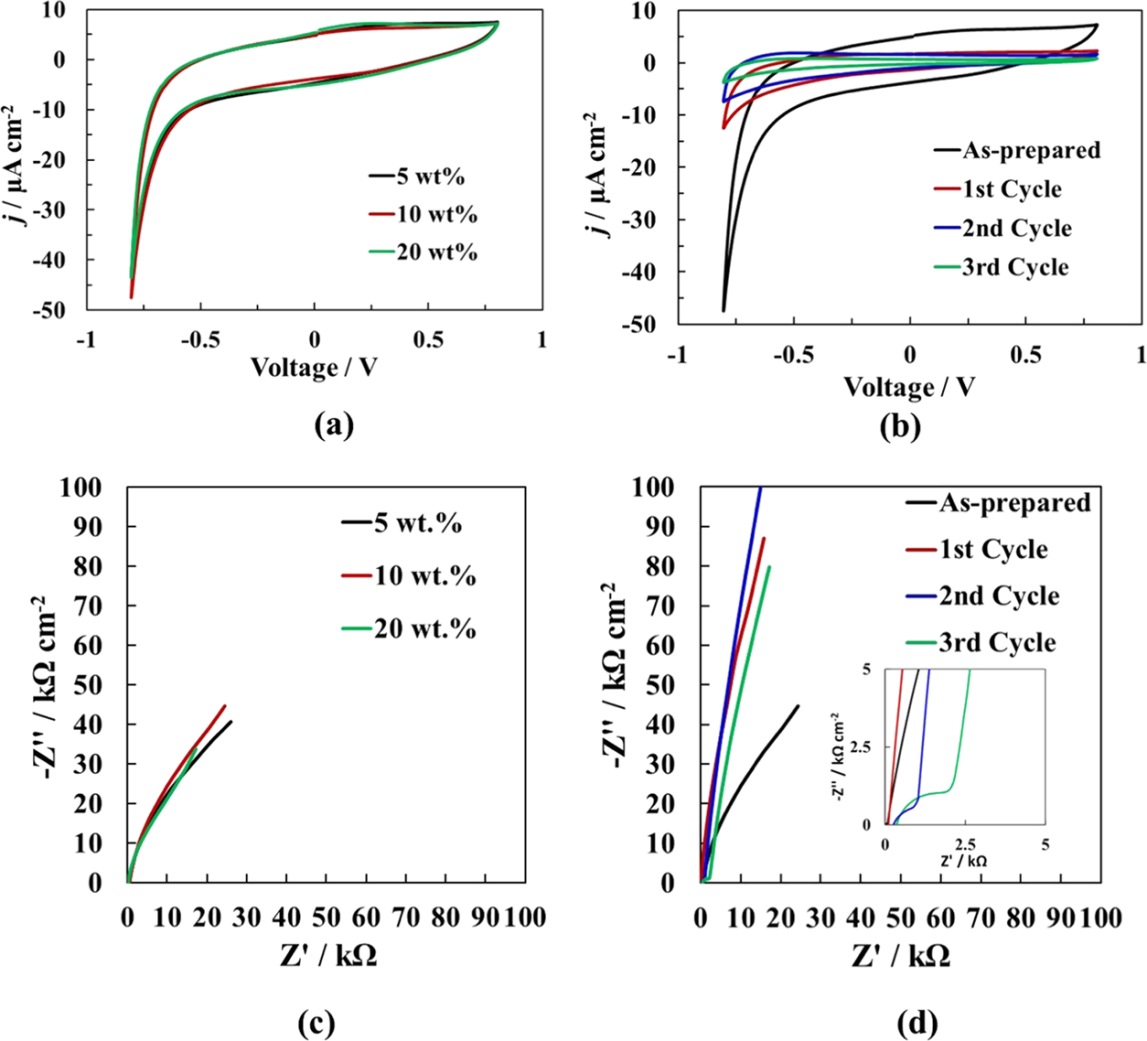
(a) Cyclic voltammograms of PEDOT/Alg/Fe_3_O_4_ hydrogels with different amounts of magnetite nanoparticles (5, 10, and 20 wt %). (b) Cyclic voltammograms of the as-prepared and rebuilt PEDOT/Alg/10 wt % Fe_3_O_4_ hydrogels up to 3 disassembly/re-cross-linking cycles. (c) EIS spectra of PEDOT/Alg/Fe_3_O_4_ hydrogels with different amounts of magnetite nanoparticles (5, 10, and 20 wt %). (d) EIS spectra of the as-prepared and rebuilt PEDOT/Alg/10 wt % Fe_3_O_4_ hydrogels up to 3 disassembly/re-cross-linking cycles.

The electrochemical properties of both as-prepared and rebuilt hydrogels were also characterized using cyclic voltammetry (CV) and electrochemical impedance spectroscopy (EIS). Figure 6a shows the CV of the PEDOT/Alg/Fe_3_O_4_ hydrogels with different magnetite loadings. As can be observed, all of the voltammograms exhibited similar qualities. An increment in the reduction current was recorded beyond −0.5 V, which was mainly attributed to the hydrogen evolution reaction and to the reduction of Fe^3+^ to Fe^2+^. Moreover, a slight oxidation band was observed centered at 0.2 V, probably due to the oxidation of Fe^2+^ to Fe^3+^. Consequently, we performed the same CV experiments with the rebuilt samples (Figure 6b). After three disassembly/re-cross-linking cycles, we observed that the shape of the voltammograms was similar; however, the intensity decreased after each cycle. This result could be explained by taking into account a number of contributing phenomena. First, and as we have previously shown, the rebuilt hydrogels are more compact and show lower porosities than the as-prepared samples, which implies a lower active surface area; therefore, as the adsorption–desorption of ions is surface-dependent, a lower intensity in the CV is recorded. Second, during the rebuilding process, we observed a decrease in the PEDOT content, which reduces the electrical conductivity of the hydrogel, and hence, a lower intensity was obtained in the CV.

EIS was also performed for the as-prepared and rebuilt hydrogels to investigate their electrical resistivity. Figure 6c shows the EIS spectra of the as-prepared hydrogels with different amounts of magnetite. The similar qualities observed of the spectra indicated that increasing magnetite loadings did not significantly alter the conductivity/resistivity of the hydrogel. The Nyquist plots mainly consisted of a straight line in the low-frequency region. No semicircles were observed indicating that the charge-transfer resistance at the interface (*R*_ct_) was very low and significantly lower than the ohmic resistance. In other words, the hydrogels showed appropriate ionic conductivity, independent of the magnetite content. On the other hand, the linear dependence between *Z*″ and *Z*′ at lower frequencies, and with an angle higher than 45°, was indicative of the rapid ion transport between the hydrogel and the electrolyte. The EIS spectra of the cross-linked/disassembled hydrogels are shown in Figure 6d. While the Nyquist plots for the as-prepared and one-cycle rebuilt hydrogel did not show a semicircle, meaning low *R*_ct_ values, the two- and three-cycle rebuilt hydrogels began to develop a semicircle, indicative of an increase in the charge transfer resistance. This was likely a consequence of the loss of PEDOT during the rebuilding process (see the inset in Figure 6d), in agreement with the results of CV.

#### Magnetic Characterization

**Figure 7:**
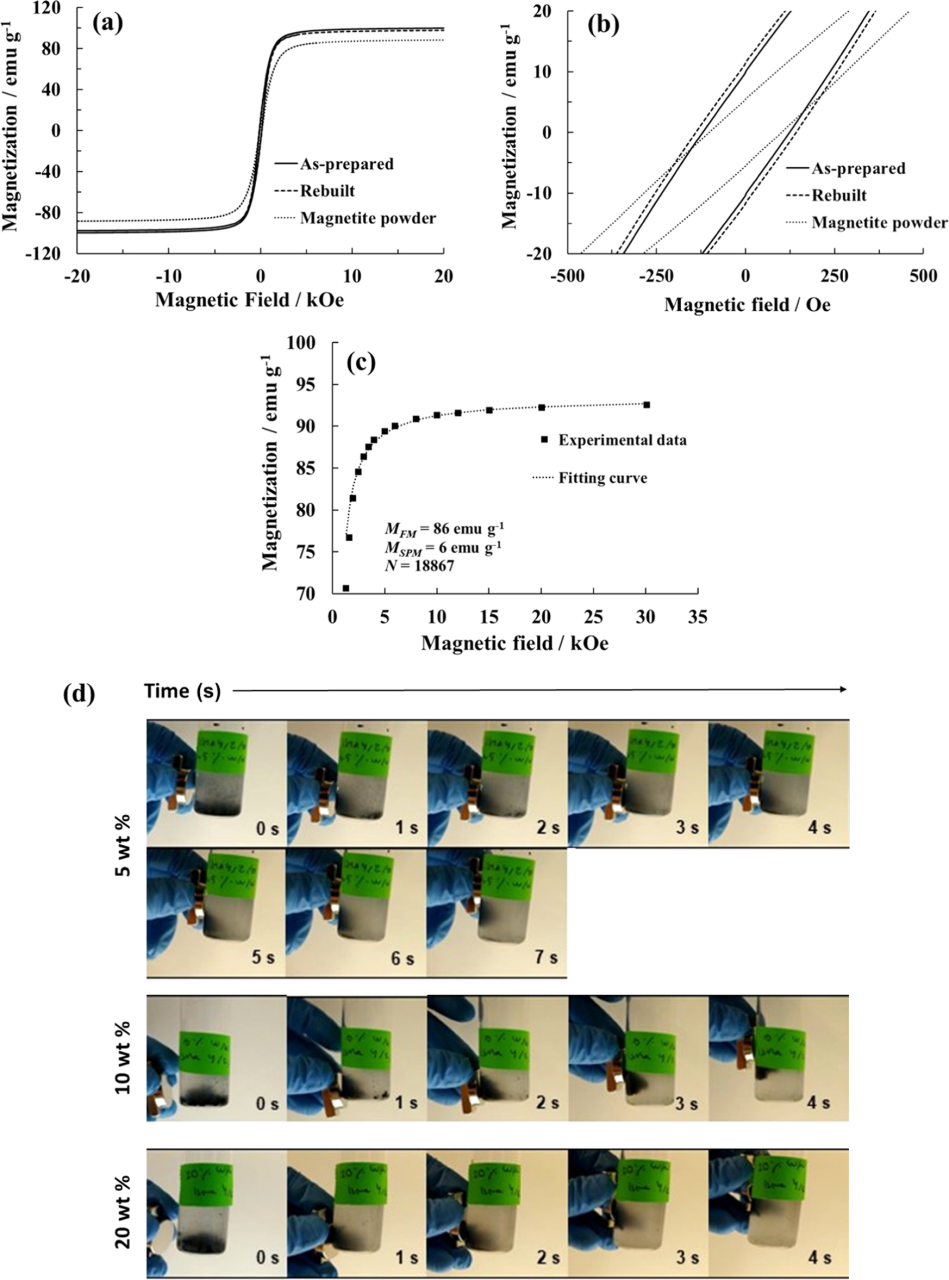
(a) *M*–*H* curves and (b) expanded view at *H* = 0 Oe for the magnetite powder, the as-prepared and three-cycle rebuilt PEDOT/Alg/10 wt % Fe_3_O_4_ hydrogels. (c) Magnetization data for the magnetite powder sample and fitting curve according to eq 5 for *H* > 1.7 kOe. The fitting parameters are displayed. (d) The sequence of images showing the response time of the polymer–Fe_3_O_4_ NP aggregates to a FeNdB permanent magnet for different amounts of magnetite (5, 10, and 20 wt %).

The magnetic properties of the hydrogels were characterized by recording the hysteresis loops using a SQUID magnetometer. Figure 7a shows the magnetization–magnetic field (*M*–*H*) curves for Fe_3_O_4_ NPs (powder), the PEDOT/Alg/10 wt % Fe_3_O_4_ hydrogel, and the three-cycle rebuilt PEDOT/Alg/10 wt % Fe_3_O_4_ hydrogel normalized with respect to the magnetite content. The saturation magnetization (*M*_s_) value for the magnetite powder was approximately the same (92 emu g^–1^) as those obtained for the hydrogels (98 and 96 emu g^–1^ for the as-prepared and the rebuilt hydrogel, respectively). The small difference can be explained by partial aggregation of NPs within the hydrogel: the higher the effective particle size, the higher the *M*_s_.^45^ Despite the difference in *M*_s_, these values are within the theoretical bulk value range reported for magnetite (92–100 emu g^–1^), indicating that its incorporation into the polymeric network did not hinder the magnetic response.^47^ Another important parameter obtained from the *M*–*H* curves is the coercive field (*H*_c_), which is indicative of the soft or hard magnetic behavior of the material. Figure 7b shows an expanded view of the hysteresis loop at around *H* = 0 Oe, where coercive fields of 120 Oe, 152 Oe, and 153 Oe were measured for the powder Fe_3_O_4_ NPs, the as-prepared, and the rebuilt hydrogels, respectively. Again, the slightly higher *H*_c_ values are explained by nanoparticle aggregation within the hydrogels.^45^ The low *H*_c_ values indicate that the magnetite nanoparticles and the hydrogels are soft magnetic. This soft character is helpful for the transport of the polymer–Fe_3_O_4_ aggregates since Fe_3_O_4_ NPs will easily change their magnetic moments with the applied magnetic field, meaning that they will show a time-dependent response. The remanent magnetization (*M*_r_) measured in the *M*–*H* loops was around 5 and 10 emu g^–1^ for the powder Fe_3_O_4_ NPs and hydrogels, respectively (Figure 7b). The low remanence values indicate that most of the magnetic moments are misaligned and practically no persistent magnetization remains once the applied field is removed. Thus, they can be considered as noninteracting particles, partially hindering NPs aggregation. Such behavior is again an advantage in having well-dispersed NPs in the cross-linked and disassembled hydrogels, as we observed in the results of EDX analysis. Comparing the *M*_s_, *M*_r_, and *H*_c_ values obtained in this work with those reported by Li et al.,^45^ it appeared that all three measured values were coherent, with particle sizes in the range 150–200 nm, which was the same size range observed by electron microscopy (Figure 2).

Although composite hydrogels demonstrated an appropriate magnetic field response, the *M*–*H* curves saturated at magnetic fields of around 5 kOe. This value is slightly higher than those reported for ferromagnetic materials and could be indicative of the presence of superparamagnetic particles, as larger magnetic fields are needed to align their magnetic moments.^53,54^ The analysis of the magnetic field dependence of the magnetization can shed light on this observation due to the differences in the mathematical expressions that govern the ferromagnetic and superparamagnetic behaviors. While the magnetic field dependence of the *M*(*H*) curve of noninteracting superparamagnetic particles can be described by the Langevin function *L*(*x*),^53,54^ the *M*(*H*) curves of samples containing both ferromagnetic and superparamagnetic particles can be described by the following expression (eq 5) for *H* > *H*_s_ (where *H*_s_ = 1.7 kOe is the saturation field of ferromagnetic particles)^53,54^
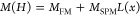
5where *M*_FM_ and *M*_SPM_ account for the room-temperature saturation magnetization values for the ferromagnetic and superparamagnetic contributions, respectively. *L*(*x*) is the Langevin function, where *x* = μ*H*/*kT* with μ being the average magnetic moment of the superparamagnetic particles. The magnetic moment μ is also expressed as μ = *N*μ_B_, where μ_B_ is the Bohr magneton and *N* is a parameter that characterizes the average macrospin of the particles. Figure 7c shows the fit of the experimental data (above *H*_s_) with eq 5 along with *M*_FM,_*M*_SPM_, and *N* parameters. A good agreement between the fitting and the experimental data was obtained. The values of *M*_FM_ and *M*_SPM_ parameters for the Fe_3_O_4_ NP powder indicate that most of the magnetization corresponds to the ferromagnetic particles (*M*_FM_ = 86 emu g^–1^); however, there is a small contribution of superparamagnetic particles (*M*_SPM_ = 6 emu g^–1^), representing only around 7% of the total magnetization. These results indicate that the magnetite nanoparticles are mostly ferromagnetic, albeit with a small number of superparamagnetic particles, which explains the nonsaturating behavior of the *M*(*H*) curve up to 5 kOe. The mean magnetic moment size was around 18 887μ_B_, which corresponds to a mean particle size of 7 nm (considering spherical particles). The same quantitative results were obtained for the as-prepared and rebuilt PEDOT/Alg/Fe_3_O_4_ hydrogels, in terms of ferromagnetic and superparamagnetic contribution. These results further demonstrate that the magnetic properties of the Fe_3_O_4_ nanoparticles were unaltered by the presence of the polymeric chains. Overall, all of the determined values are coherent with the soft ferromagnetic character of magnetite, both in powder form and within the hydrogel. Moreover, most of the magnetization contribution comes from the ferromagnetic particles rather than the superparamagnetic particles, indicating that there is a homogeneous magnetic response to an applied external magnetic field.

Finally, while taking advantage of the disassembly/re-cross-linking ability of PEDOT/Alg hydrogels, we studied the transportability of the polymer–Fe_3_O_4_ NP aggregates obtained after disassembly of the hydrogels by assessing their response to a magnet. When a magnet was placed on the vial wall, the aggregates were clearly attracted toward the magnet. The time lapse was measured, and while no differences were observed between 10 and 20 wt %, we detected that the mixture containing 5 wt % magnetite was transported at lower velocity, which was attributed to the low concentration of the magnetic particles (Figure 7d).

#### Spatiotemporal Control of the Hydrogel Using Magnetic Fields

**Figure 8:**
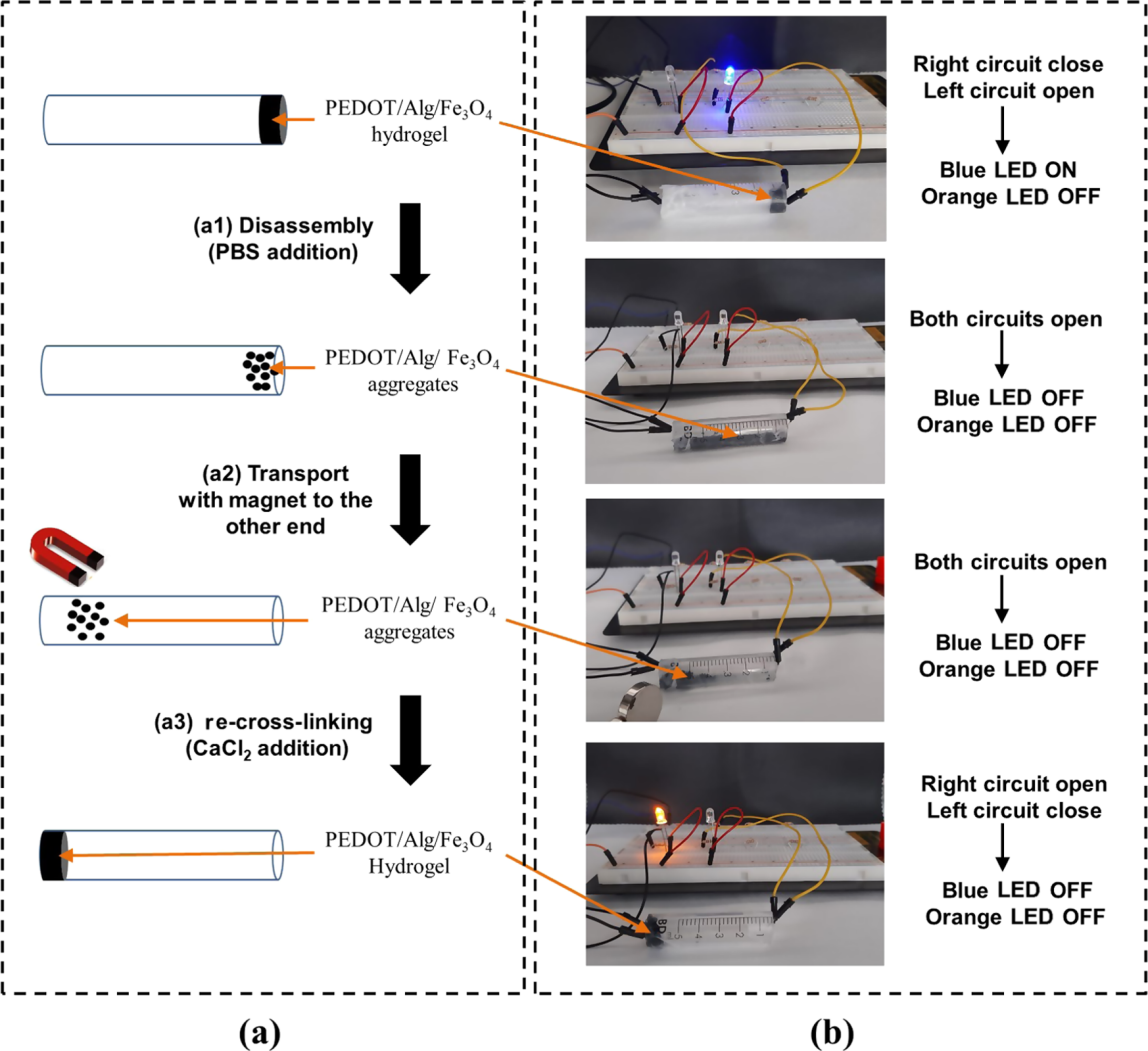
(a) Scheme showing the strategy followed for the magnetic transport of the hydrogel: (a1) disassembly of the hydrogel by the addition of PBS solution, (a2) transport of the aggregates to the other end of the tube, and (a3) re-cross-linking of the hydrogel by the addition of CaCl_2_ solution. (b) Sequence of images showing the steps schematically shown in part (a). Each end of the tube was connected to an external circuit containing a LED. The LED on the right circuit is blue and the LED on the left circuit is orange. The LEDs are ON and OFF when the hydrogel is cross-linked (circuits closed) and disassembled (circuits open), respectively.

As a proof of concept, we assessed the magnetic transportability of the PEDOT/Alg/10 wt % Fe_3_O_4_ hydrogel in a dynamic and continuous manner following four different steps (Figure 8a). First, we prepared the hydrogel at one end of a cylindrical tube by introducing the dispersion and adding a CaCl_2_ solution to cross-link the system. After that, CaCl_2_ was replaced by PBS solution to disassemble the hydrogel. Then, the polymer–Fe_3_O_4_ NP aggregates were magnetically transported to the other end of the tube using a FeNdB permanent magnet. Finally, the hydrogel was re-cross-linked by adding CaCl_2_ solution. The same four steps are displayed in the sequence of images taken during the experiment (Figure 8b), corroborating a successful transport of the hydrogel using a magnetic field. To rapidly check the functionality of the hydrogel before and after the magnetic transport, each end of the tube was connected to an external circuit containing a light-emitting diode (LED). At the beginning of the experiment, when the hydrogel was on the right-hand side of the tube, the LED (blue) was ON because the electrically conductive hydrogel closed the circuit. Meanwhile, the LED (orange) on the left circuit was OFF as the circuit was open. Then, when the hydrogel was disassembled, the electrical conductivity was lost because the PEDOT-containing aggregates were not in contact and therefore lacked an electronic path. Thus, both LEDs were OFF as the circuits were open. Finally, once the hydrogel was transported to the left side of the tube and re-cross-linked, the orange LED was ON, indicating that the electrical conductivity of the hydrogel was recovered; the blue LED was OFF as the circuit was then open.

#### Viability of the Dual Hydrogel for Temperature Sensing and Magnetic Hyperthermia and Its Transportability

**Figure 9:**
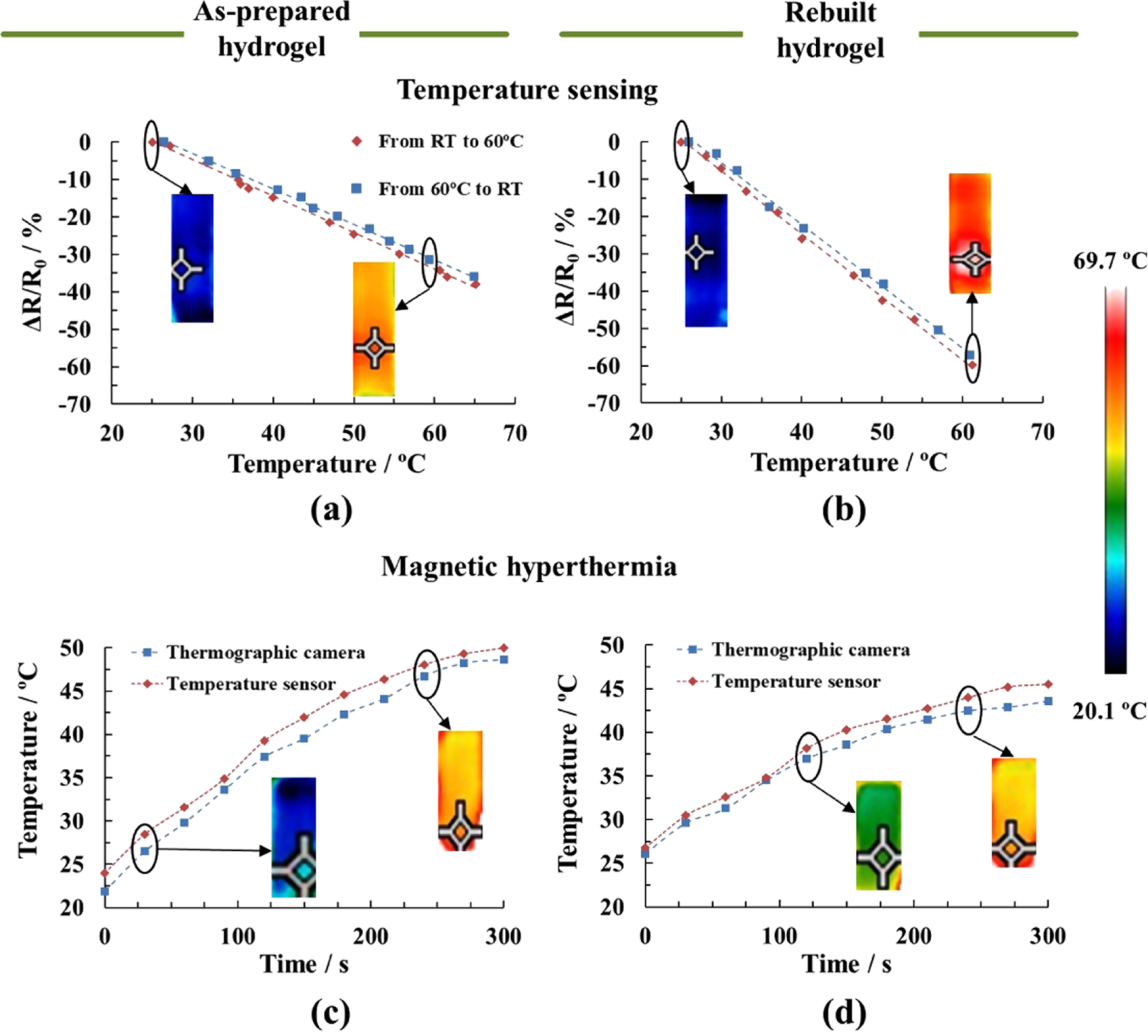
(a, b) Normalized resistance change as a function of temperature for the as-prepared and three-cycle rebuilt hydrogel. (c, d) Variation of temperature as a function of magnetic field application time for the as-prepared and three-cycle rebuilt hydrogel. Insets show images taken with the thermographic camera at different temperatures or time sets.

Having demonstrated the hydrogel spatiotemporal control performance and recovery of the electrical properties after transport, we subsequently evaluated two different applications of the multifunctional hydrogel: temperature sensing and magnetic hyperthermia. These are just two of the wide range of applications that can be proposed for such dual stimuli-responsive hydrogels. While PEDOT is a thermoresistor material that allows sensing the variation of resistance with temperature,^55,56^ magnetite nanoparticles generate heat when exposed to high-frequency alternating magnetic fields. First, the PEDOT/Alg/10 wt % Fe_3_O_4_ hydrogel was evaluated as a temperature sensor by measuring the variation of the normalized resistance (Δ*R*/*R*_0_) by changing the temperature from ambient conditions (∼25 °C) to 60 °C and vice versa (Figure 9a). As can be observed, the normalized resistance decreased with temperature because the electrical conductivity of PEDOT increased. This is because PEDOT is a semiconducting material and an increase in temperature enhances the charge carrier transport as well as the generation of charge carriers.^55,56^ From this data, the temperature coefficient of resistance (TCR) can be calculated (eq 6), which defines the sensitivity of the hydrogel
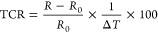
6The sensitivity was −1.35 ± 0.15% °C^–1^, which is slightly higher than the values reported for similar temperature sensors based on PEDOT.^55,56^ On the other hand, a practically negligible hysteresis was observed after decreasing from 60 °C to room temperature, showing the reproducibility of the hydrogel sensor. The use of a thermographic camera evidenced that the entire hydrogel acquired the same temperature due to a homogeneous distribution of the electrically conducting PEDOT (see images in Figure 9a). These results are in agreement with those previously shown by EDX and ATR-FTIR analyses. After that, we evaluated the performance of the three-cycle re-cross-linked hydrogel, observing the same response and tendency as for the as-prepared samples (Figure 9b). However, the TCR (−1.78 ± 0.18% °C^–1^) was slightly higher than that of the as-prepared hydrogel, which was attributed to the loss of PEDOT after 3 disassembly/re-cross-linking cycles, making the hydrogel more resistive. The thermographic images showed again a homogeneous temperature distribution throughout the entire re-cross-linked hydrogel (see images in Figure 9b).

We also evaluated the capacity of the hydrogel to generate heat for magnetic hyperthermia applications. To do so, the as-prepared and rebuilt hydrogels were placed in a solenoid coil and were subjected to an alternating magnetic field (200 kHz, 8 kA m^–1^). The temperature was simultaneously measured using a thermographic camera and the temperature sensing capacity of the PEDOT/Alg/Fe_3_O_4_ hydrogel, as previously shown in Figure 9a,b. As can be observed in Figure 9c,d, there was an increase in temperature as the magnetic induction heating time increased up to an almost constant temperature. It is important to highlight that the temperatures measured from both methodologies followed the same trend with similar values; at least within the error of the thermographic camera used (±3 °C), indicating the excellent dual performance of the hydrogel for magnetic hyperthermia and simultaneous in situ temperature sensing. On the other hand, the temperature reached by the as-prepared and three-cycle rebuilt hydrogels, after applying the magnetic field for 5 min, was around 49 and 45 °C, respectively. The induced heat can be explained by the following two mechanisms: (i) the eddy current due to the electrical conductivity of the hydrogel and (ii) the hysteresis loss, which has been reported effective for magnetic particles with sizes larger than 100 nm.^57^ Therefore, the change in the plateau temperature can be attributed to the loss of PEDOT and Alg from the hydrogel during re-cross-linking and thus the increase in the relative content of the magnetite NPs. The thermographic images taken at different times revealed that the heat was homogeneously induced, rather than locally, throughout the entire hydrogel. Thus, both mechanisms ensure a fairly good distribution of the induced heat since the entire hydrogel was still electrically conductive and the Fe_3_O_4_ NPs remained homogeneously distributed.

The results presented in this work are promising and open the door to a myriad of applications, not only at the macro-, but also at the micro/nanoscale, mainly in the biomedical field. For example, transporting multifunctional conductive hydrogels remotely (e.g., magnetic fields) through narrow channels can be of special relevance for (i) disease theranostics: treating diseases (e.g., cancer, neuronal, or circulatory system disorders) while simultaneously measuring in situ clinically relevant parameters (e.g., temperature, pressure, biomarkers, etc.); (ii) tissue engineering applications: inducing tissue growth by magnetic and electric stimulation and sensing cell growth by electrical measurements; or (iii) drug delivery systems: inducing on-demand drug delivery via physical stimuli (e.g., electrical, magnetic, temperature, etc.) while measuring clinically relevant parameters. Nonetheless, there are several challenges of this technology to be addressed to improve the performance of the hydrogels. For example, magnetite nanoparticles could be covalently functionalized with the alginate and/or PEDOT chains to avoid their loss after each disassembly/re-cross-linking cycle, thus enhancing the number of cycles the hydrogel-based devices can be employed. Also, the utilization of hydrogels where reversible cross-linking is induced by physical (e.g., light, temperature) rather than chemical stimuli, would provide a technology more compatible with biological systems for biomedical applications.

## Conclusions

We have successfully fabricated hydrogels with magnetic and electrical performances integrated into the same system, which can be used as a novel and effective strategy to spatiotemporally transport the hydrogel using magnetic fields. The hydrogel is composed of an interpenetrated polymer network, which consists of PEDOT and Alg, conferring electrical and mechanical properties to the hydrogel, respectively. Meanwhile, magnetite nanoparticles are embedded into the hydrogel to endow it with magnetic and magnetothermal responses, which favor the entanglement of the polymeric network. The remote transport of the hydrogel was successfully achieved taking advantage of the disassembly/re-cross-linking ability of alginate and the magnetic properties of magnetite. Once the hydrogel was disassembled, the polymer–Fe_3_O_4_ NP aggregates were directionally propelled via magnetic fields and able to navigate through places, in which the cross-linked hydrogel would not pass. Once at the new target, the aggregates were re-cross-linked to rebuild the multifunctional hydrogel. The multifunctionality was further demonstrated by simultaneously using the hydrogel for temperature sensing and magnetic hyperthermia. Although in the present study we could only repeat the disassembly/re-cross-linking cycle up to three times, we believe this strategy will set a precedent to fabricate novel intelligent hydrogels with a myriad of applications in biotechnology.

## Experimental Section

### Materials

Alginic acid (molecular weight, *M*_w_ = 240 kDa), PEDOT:PSS suspension (1.3 wt %), PBS solution, and Fe_3_O_4_ NPs were obtained from Sigma Aldrich. Calcium chloride (CaCl_2_) was purchased from Scharlab. Milli-Q water grade (0.055 S cm^–1^) was used in all solutions and synthetic processes.

### Synthesis of PEDOT/Alg/Fe_3_O_4_ Hydrogels

In this study, PEDOT/Alg/Fe_3_O_4_ hydrogels were fabricated following a simple process, which consists of mixing commercial PEDOT:PSS, alginic acid, and magnetite nanoparticles and further cross-linking with CaCl_2_. First, a suspension of PEDOT:PSS/Alg at a 1:3 ratio was prepared by mixing equal volumes of a PEDOT:PSS suspension (1.3 wt %) with an alginate solution (3.9 wt %). The dispersion was magnetically stirred for 20 min at room temperature to assure a complete homogenization between both components. After that, different amounts of magnetite powder were added to the previous dispersion to obtain hydrogels with 5, 10, and 20 wt % Fe_3_O_4_ NPs (referred to the weight of the solid materials). The dispersion was sonicated in an ultrasound bath and immediately poured into a silicone mold of 2 × 0.4 × 0.5 cm^3^. Finally, the mold was immersed in a CaCl_2_ solution (3 wt %) to cross-link the alginate. The dispersion was allowed to gel at room temperature. PEDOT/Alg hydrogels were also prepared for comparison purposes. They were prepared following the protocol previously described without the addition of magnetite nanoparticles.

### Characterization

Hydrogel surface morphology was evaluated using a Zeiss Neon 40 analytical field emission scanning electron microscope (FESEM). Samples were first lyophilized and then mounted on a stainless steel stub. The applied voltage for observation was 5 kV. EDX analyses were performed with the same scanning electron microscope to study the distribution of the different components (e.g., alginate, PEDOT, and Fe_3_O_4_ NPs). The hydrogel pore size was determined from the SEM images using software SmartTIFF (v1.0.1.2.). The size and morphology of the nanoparticles were studied by transmission electron microscopy (TEM) using a JEOL 2100 microscope working at 200 kV. The structure of the nanoparticles was determined by high-resolution transmission electron microscopy (HRTEM) combined with fast Fourier transform (FFT). Samples were prepared by casting a drop of the nanoparticle dispersion onto a carbon-coated copper grid sample holder followed by evaporation at room temperature.

Attenuated total reflectance Fourier transform infrared spectroscopy (ATR-FTIR) spectra were obtained with an FTIR 4100 spectrophotometer, equipped with a diamond crystal (Specac model MKII Golden Gate Heated Single Reflection Diamond ATR). The samples were evaluated using spectra manager software. For each sample, 3 scans were performed between 4000 and 500 cm^–1^ with a resolution of 4 cm^–1^ at room temperature.

Rheological measurements of the hydrogels were carried out using a rotational rheometer (TA Instruments). All rheology measurements were made by placing the hydrogels onto the lower serrated plate (diameter: 20 mm) and the upper plate was lowered to a 1 mm gap distance. Storage modulus (*G*′) and loss modulus (*G*″) were measured using the oscillatory time sweep mode at a frequency of 1 Hz and a displacement of 1 × 10^–3^ rad. The measurements were performed at room temperature. The experiments were performed in triplicate, and the data represents the average.

Mechanical properties were evaluated by performing 20 imprints heterogeneously distributed along the specimens using a Nano Indenter XP System (MTS) with continuous stiffness measurement (CSM) having a harmonic displacement of 2 nm and a constant frequency of 45 Hz. The goal of these tests is to obtain the indentation stress–strain curve and the main characteristic parameters (mean contact pressure *p*_m_, yield strength σ_ys_, and maximum tensile and shear stress σ_tm_ and τ_max_, respectively) for porous polymeric materials. The strain rate along the indentation tests is held constant at 0.05 s^–1^. Spherical indentation tests were performed at room temperature using an indenter of a tip radius of 1 μm, and at a maximum displacement into a surface of 1000 nm.

Electrochemical measurements (CV and EIS) were performed at room temperature using a microcomputer-controlled potentiostat/galvanostat Autolab with the PGSTAT101 equipment and Nova software. A conventional three-electrode cell was used with the conducting hydrogel supported onto an ITO-coated PET sheet as a working electrode, Ag/AgCl/KCl (3 M) as a reference electrode, and a platinum wire as a counter electrode. The electrolyte employed was 0.1 M CaCl_2_ solution. In the CV experiments, the potential was scanned from 0.0 to 0.8 V at a scan rate of 100 mV s^–1^. EIS measurements were performed between 10^–1^ and 10^4^ Hz frequencies using a previously reported through-plane impedance cell.^58^

The magnetic properties of the magnetite powder and hydrogel samples were measured in a SQUID magnetometer at room temperature in a helium atmosphere. The magnetization–magnetic field curves were recorded maintaining the samples parallel to the applied magnetic field. Saturation magnetization values were given in emu g^–1^, where g is the total weight of the magnetite nanoparticles in the powder or the hydrogel. Transportability experiments were performed analyzing the response time of the de-cross-linked hydrogel to a FeNdB hard magnet.

The resistance change of the hydrogel with temperature was monitored by a digital multimeter (PeakTech 2005) by the two-point probe method. A hotplate was used to heat the sample using a thermocouple to control the temperature. A thermal imaging camera (GTC 400 C, Bosch) was also used to observe the distribution of temperature in the hydrogel. Magnetic hyperthermia experiments were performed with a homemade setup by placing the sample into the solenoid coil and applying an alternating magnetic field with a frequency of 200 kHz and a field strength of 10 kA m^–1^. The temperature was measured with time using the change in the normalized resistance of the hydrogel itself and also using the thermographic camera.

## Supporting Information Available

Information about TEM and HRTEM images of Fe_3_O_4_ NPs; the SEM image of PEDOT/Alg hydrogel; scheme of the disassembly and re-cross-linking process of the hydrogel; variation of the composition of hydrogels; evaluation of *G*′ and *G*″ moduli (PDF)

## References

[ref1] Griffin D. R.; Weaver W. M.; Scumpia P. O.; Carlo D. D.; Segura T. Accelerated Wound Healing by Injectable Microporous Gel Scaffolds Assembled from Annealed Building Blocks. Nat. Mater. 2015, 14, 737–744. 10.1038/nmat4294. 26030305 PMC4615579

[ref2] Jayawarna W.; Ali M.; Jowitt T. A.; Miller A. F.; Saianni A.; Gough J. E.; Ulijn R. V. Nanostructured Hydrogels for Three-Dimensional Cell Culture through Self-Assembly of Fluorenylmethoxycarbonyl–Dipeptides. Adv. Mater. 2006, 18, 611–614. 10.1002/adma.200501522.

[ref3] Vegas A. J.; Veiseh O.; Doloff J. C.; Ma M.; Tam H. H.; Bratlie K.; Li J.; Bader A. R.; Langan E.; Olejnik K.; Fenton P.; Kang J. W.; Hollister-Locke J.; Bochenek M. A.; Chiu A.; Siebert S.; Tang K.; Jhunjhunwala S.; Aresta-Dasilva S.; Dholakia N.; Thakrar R.; Vietti T.; Chen M.; Cohen J.; Siniakowicz K.; Qi M.; McGarrigle J.; Graham A. C.; Lyle S.; Harlan D. M.; Greiner D. L.; Oberholzer J.; Weir G. C.; Langer R.; Anderson D. G. Combinatorial Hydrogel Library Enables Identification of Materials that Mitigate the Foreign Body Response in Primates. Nat. Biotechnol. 2016, 34, 345–352. 10.1038/nbt.3462. 26807527 PMC4904301

[ref4] Lee K. Y.; Mooney D. J. Hydrogels for Tissue Engineering. Chem. Rev. 2001, 101, 1869–1880. 10.1021/cr000108x. 11710233

[ref5] Zhang Y. S.; Khademhosseini A. Advances in Engineering Hydrogels. Science 2017, 356, eaaf3627–eaaf3636. 10.1126/science.aaf3627. 28473537 PMC5841082

[ref6] Liu X.; Liu J.; Lin S.; Zhao X. Hydrogel Machines. Mater. Today 2020, 36, 102–124. 10.1016/j.mattod.2019.12.026.

[ref7] Yuk H.; Lu B.; Zhao X. Hydrogel Bioelectronics. Chem. Soc. Rev. 2019, 48, 1642–1667. 10.1039/C8CS00595H. 30474663

[ref8] Shastri A.; M.McGregor L.; Liu Y.; Harris V.; Nan H.; Mujica M.; Vasquez Y.; Bhattacharya A.; Ma Y.; Aizenberg M.; Kuksenok O.; Balazs A. C.; Aizenberg J.; He X. An Aptamer-Functionalized Chemomechanically Modulated Biomolecule Catch-and-Release System. Nat. Chem. 2015, 7, 447–454. 10.1038/nchem.2203. 25901824

[ref9] Guo J.; Liu X.; Jiang N.; Yetisen A. K.; Yuk H.; Yang C.; Khademhosseini A.; Zhao X.; Yun S.-H. Highly Stretchable, Strain Sensing Hydrogel Optical Fibers. Adv. Mater. 2016, 28, 10244–10249. 10.1002/adma.201603160. 27714887 PMC5148684

[ref10] Lu B.; Yuk H.; Li S.; Jian N.; Qu K.; Xu J.; Zhao X. Pure PEDOT:PSS Hydrogels. Nat. Commun. 2019, 10, 1043 10.1038/s41467-019-09003-5. 30837483 PMC6401010

[ref11] Lee Y. Y.; Kang H.-Y.; Gwon S. H.; Choi G. M.; Lim S.-M.; Sun J.-Y.; Joo Y.-C. A Strain-Insensitive Stretchable Electronic Conductor: PEDOT:PSS/Acrylamide Organogels. Adv. Mater. 2016, 28, 1636–1643. 10.1002/adma.201504606. 26684678

[ref12] Huang H.-W.; Uslu F. E.; Katsamba P.; Lauga E.; Sakar M. S.; Nelson B. J. Adaptive Locomotion of Artificial Microswimmers. Sci. Adv. 2017, 2, eaau1532 10.1126/sciadv.aau1532. PMC6357760 30746446

[ref13] Araújo-Custódio S.; Gomez-Florit M.; Tomas A. R.; Mendes B. B.; Babo P. S.; Mithieux S. M.; Weiss A.; Domingues R. M. A.; Reis R. L.; Gomes M. E. Injectable and Magnetic Responsive Hydrogels with Bioinspired Ordered Structures. ACS Biomater. Sci. Eng. 2019, 5, 1392–1404. 10.1021/acsbiomaterials.8b01179. 33405615

[ref14] Lanzalaco S.; Turon P.; Weis C.; Mata C.; Planas E.; Aleman C.; Armelin E. Toward the New Generation of Surgical Meshes with 4D Response: Soft, Dynamic, and Adaptable. Adv. Funct. Mater. 2020, 30, 2004145 10.1002/adfm.202004145.

[ref15] Choi M.; Humar M.; Kim S.; Yun S.-H. Step-Index Optical Fiber Made of Biocompatible Hydrogels. Adv. Mater. 2015, 27, 4081–4086. 10.1002/adma.201501603. 26045317 PMC4503511

[ref16] Mawad D.; Artzy-Schnirman A.; Tonkin J.; Ramos J.; Inal S.; Mahat M. M.; Darwish N.; Zwi-Dantsis L.; Malliaras G. G.; Gooding J. J.; Lauto A.; Stevens M. M. Electroconductive Hydrogel Based on Functional Poly(ethylenedioxy thiophene). Chem. Mater. 2016, 28, 6080–6088. 10.1021/acs.chemmater.6b01298. 27656042 PMC5024651

[ref17] Wang L.; Xu T.; Zhang X. Multifunctional Conductive Hydrogel-Based Flexible Wearable Sensors. TrAC, Trends Anal. Chem. 2021, 134, 116130–116141. 10.1016/j.trac.2020.116130.

[ref18] Shin S. R.; Jung S. M.; Zalabany M.; Kim K.; Zorlutuna P.; Kim S. B.; Nikkhah M.; Khabiry M.; Azize M.; Kong J.; Wan K.-T.; Palacios T.; Dokmeci M. R.; Bae H.; Tang X.; Khademhosseini A. Carbon-Nanotube-Embedded Hydrogel Sheets for Engineering Cardiac Constructs and Bioactuators. ACS Nano 2013, 7, 2369–2380. 10.1021/nn305559j. 23363247 PMC3609875

[ref19] Xu Y.; Cui M.; Patsis P. A.; Günther M.; Yang X.; Eckert K.; Zhang Y. Reversibly Assembled Electroconductive Hydrogel via a Host–Guest Interaction for 3D Cell Culture. ACS Appl. Mater. Interfaces 2019, 11, 7715–7724. 10.1021/acsami.8b19482. 30714715

[ref20] Mehrali M.; Thakur A.; Pennisi C. P.; Talebian S.; Arpanaei A.; Nikkhah M.; Dolatshahi-Pirouz A. Nanoreinforced Hydrogels for Tissue Engineering: Biomaterials that are Compatible with Load-Bearing and Electroactive Tissues. Adv. Mater. 2017, 29, 1603612 10.1002/adma.201603612. 27966826

[ref21] Zhu K.; Shin S. R.; Van Kempen T.; Li Y. C.; Ponraj V.; Nasajpour A.; Mandla S.; Hu N.; Liu X.; Leijten J.; Lin Y. D.; Hussain M. A.; Zhang Y. S.; Tamayol A.; Khademhosseini A. Gold Nanocomposite Bioink for Printing 3D Cardiac Constructs. Adv. Funct. Mater. 2017, 27, 1605352 10.1002/adfm.201605352. 30319321 PMC6181228

[ref22] Navaei A.; Saini H.; Christenson W.; Sullivan R. T.; Ros R.; Nikkhah M. Gold Nanorod-Incorporated Gelatin-Based Conductive Hydrogels for Engineering Cardiac Tissue Constructs. Acta Biomater. 2016, 41, 133–146. 10.1016/j.actbio.2016.05.027. 27212425

[ref23] Wickham A.; Vagin M.; Khalaf H.; Bertazzo S.; Hodder P.; Danmark S.; Bengtsson T.; Altimiras J.; Aili D. Electroactive Biomimetic Collagen-Silver Nanowire Composite Scaffolds. Nanoscale 2016, 8, 14146–14155. 10.1039/C6NR02027E. 27385421

[ref24] Shin S. R.; Aghaei-Ghareh-Bolagh B.; Gao X. G.; Nikkhah M.; Jung S. M.; Dolatshahi-Pirouz A.; Kim S. B.; Kim S. M.; Dokmeci M. R.; Tang X. W.; Khademhosseini A. Layer-by-Layer Assembly of 3D Tissue Constructs with Functionalized Graphene. Adv. Funct. Mater. 2014, 24, 6136–6144. 10.1002/adfm.201401300. 25419209 PMC4235968

[ref25] Mohammadi Amirabad L. M.; Massumi M.; Shamsara M.; Shabani I.; Amari A.; Mohammadi M. M.; Hosseinzadeh S.; Vakilian S.; Steinbach S. K.; Khorramizadeh M. R.; Soleimani M.; Barzin J. Enhanced Cardiac Differentiation of Human Cardiovascular Disease Patient-Specific Induced Pluripotent Stem Cells by Applying Unidirectional Electrical Pulses Using Aligned Electroactive Nanofibrous Scaffolds. ACS Appl. Mater. Interfaces 2017, 9, 6849–6864. 10.1021/acsami.6b15271. 28116894

[ref26] Zhang W.; Feng P.; Chen J.; Sun Z.; Zhao B. Electrically Conductive Hydrogels for Flexible Energy Storage Systems. Prog. Polym. Sci. 2019, 88, 220–240. 10.1016/j.progpolymsci.2018.09.001.

[ref27] Saborío M. C. G.; Lanzalaco S.; Fabregat G.; Puiggali J.; Estrany F.; Alemán C. Flexible Electrodes for Supercapacitors Based on the Supramolecular Assembly of Biohydrogel and Conducting Polymer. J. Phys. Chem. C 2018, 122, 1078–1090. 10.1021/acs.jpcc.7b10738.

[ref28] Garcia-Torres J.; Crean C. Ternary Composite Solid-State Flexible Supercapacitor Based on Nanocarbons/Manganese Dioxide/PEDOT:PSS Fibres. Mater. Des. 2018, 155, 194–202. 10.1016/j.matdes.2018.05.070.

[ref29] Reid D. O.; Smith R. E.; Garcia-Torres J.; Watts J. F.; Crean C. Solvent Treatment of Wet-Spun PEDOT: PSS Fibers for Fiber-Based Wearable pH Sensing. Sensors 2019, 19, 4213–4222. 10.3390/s19194213. PMC6806142 31569329

[ref30] Inal S.; Rivnay J.; Suiu A.-O.; Malliaras G. G.; McCulloch I. Conjugated Polymers in Bioelectronics. Acc. Chem. Res. 2018, 51, 1368–1376. 10.1021/acs.accounts.7b00624. 29874033

[ref31] Babeli I.; Ruano G.; Casanovas J.; Ginebra M.-P.; Garcia-Torres J.; Aleman C. Conductive, Self-Healable and Reusable Poly(3,4-ethylenedioxythiophene)-Based Hydrogels for Highly Sensitive Pressure Arrays. J. Mater. Chem. C 2020, 8, 8654–8667. 10.1039/D0TC01947J.

[ref32] Liu Y.; Liu J.; Chen S.; Lei T.; Kim Y.; Niu S.; Wang H.; Wang X.; Foudeh A. M.; Tok J. B.-H.; Bao Z. Soft and Elastic Hydrogel-Based Microelectronics for Localized Low-Voltage Neuromodulation. Nat. Biomed. Eng. 2019, 3, 58–68. 10.1038/s41551-018-0335-6. 30932073

[ref33] Bock N.; Riminucci A.; Dionigi C.; Russo A.; Tampieri A.; Landi E.; Goranov V. A.; Marcacci M.; Dediu V. A Novel Route in Bone Tissue Engineering: Magnetic Biomimetic Scaffolds. Acta Biomater. 2010, 6, 786–796. 10.1016/j.actbio.2009.09.017. 19788946

[ref34] Li Y.; Huang G.; Zhang X.; Li B.; Chen Y.; Lu T.; Lu T. J.; Xu F. Magnetic Hydrogels and their Potential Biomedical Applications. Adv. Funct. Mater. 2013, 23, 660–672. 10.1002/adfm.201201708.

[ref35] Kim Y.; Yuk H.; Zhao R.; Chester S. A.; Zhao X. Printing Ferromagnetic Domains for Untethered Fast-Transforming Soft Materials. Nature 2018, 558, 274–279. 10.1038/s41586-018-0185-0. 29899476

[ref36] Soares P. I. P.; Romao J.; Matos R.; Silva J. C.; Borges J. P. Design and Engineering of Magneto-Responsive Devices for Cancer Theranostics: Nano to Macro Perspective. Prog. Mater. Sci. 2021, 116, 100742 10.1016/j.pmatsci.2020.100742.

[ref37] Deng Z.; Yu R.; Guo B. Stimuli-Responsive Conductive Hydrogels: Design, Properties, and Applications. Mater. Chem. Front. 2021, 5, 2092–2139. 10.1039/D0QM00868K.

[ref38] Liu K.; Han L.; Tang P.; Yang K.; Gan D.; Wang X.; Wang K.; Ren F.; Fang L.; Xu Y.; Lu Z.; Lu X. An Anisotropic Hydrogel Based on Mussel-Inspired Conductive Ferrofluid Composed of Electromagnetic Nanohybrids. Nano Lett. 2019, 19, 8343–8356. 10.1021/acs.nanolett.9b00363. 31659907

[ref39] Li P.; Zhang S.; Li K.; Wang J.; Liu M.; Gu X.; Fan Y. The Promoting Effect on Pre-Osteoblast Growth Under Electrical and Magnetic Double Stimulation Based on PEDOT/Fe_3_O_4_/PLGA Magnetic-Conductive Bi-Functional Scaffolds. J. Mater. Chem. B 2018, 6, 4952–4962. 10.1039/C8TB00985F. 32255068

[ref40] Wang Y.; Zhu Y.; Xue Y.; Wang J.; Li X.; Wu X.; Qin Y.; Chen W. Sequential In-Situ Route to Synthesize Novel Composite Hydrogels with Excellent Mechanical, Conductive, and Magnetic Responsive Properties. Mater. Des. 2020, 193, 108759 10.1016/j.matdes.2020.108759.

[ref41] Liu Y.; He K.; Chen G.; Leow W. R.; Chen X. Nature-Inspired Structural Materials for Flexible Electronic Devices. Chem. Rev. 2017, 117, 12893–12941. 10.1021/acs.chemrev.7b00291. 28991450

[ref42] Fogelson A. L.; Hussain Y. H.; Leiderman K. Blood Clot Formation Under Flow: The Importance of Factor XI Depends Strongly on Platelet Count. Biophys. J. 2012, 102, 10–18. 10.1016/j.bpj.2011.10.048. 22225793 PMC3250692

[ref43] García-Torres J.; Calero C.; Sagues F.; Pagonabarraga I.; Tierno P. Magnetically Tunable Bidirectional Locomotion of a Self-Assembled Nanorod-Sphere Propeller. Nat. Commun. 2018, 9, 1663 10.1038/s41467-018-04115-w. 29695717 PMC5916950

[ref44] Martinez-Boubeta C.; Simeonidis K.; Makridis A.; Angelakeris M.; Iglesias O.; Guardia P.; Cabot A.; Yedra L.; Estrade S.; Peiro F.; Saghi Z.; Midgley P. A.; Conde-Leboran I.; Serantes D.; Baldomir D. Learning from Nature to Improve the Heat Generation of Iron-Oxide Nanoparticles for Magnetic Hyperthermia Applications. Sci. Rep. 2013, 3, 1652 10.1038/srep01652. 23576006 PMC3622918

[ref45] Li Q.; Kartikowati C. W.; Horie S.; Ogi T.; Iwaki T.; Okuyama K. Correlation Between Particle Size/Domain Structure and Magnetic Properties of Highly Crystalline Fe_3_O_4_ Nanoparticles. Sci. Rep. 2017, 7, 9894 10.1038/s41598-017-09897-5. 28855564 PMC5577113

[ref46] US Department of Commerce. Standard X-ray Diffraction Powder Patterns; National Bureau of Standards, 1967; Vol. 25, p 31.

[ref47] Montoya P.; Marin T.; Mejia S.; Arnache O.; Calderon J. A. Elucidation of the Mechanism of Electrochemical Formation of Magnetite Nanoparticles by In Situ Raman Spectroscopy. J. Electrochem. Soc. 2017, 164, D1056–D1065. 10.1149/2.0181802jes.

[ref48] Puiggalí-Jou A.; Cazorla E.; Ruano G.; Babeli I.; Ginebra M.-P.; Garcia-Torres J.; Aleman C. Electroresponsive Alginate-Based Hydrogels for Controlled Release of Hydrophobic Drugs. ACS Biomater. Sci. Eng. 2020, 6, 6228–6240. 10.1021/acsbiomaterials.0c01400. 33449669

[ref49] Yang K.; Peng H.; Wen Y.; Li N. Re-Examination of Characteristic FTIR Spectrum of Secondary Layer in Bilayer Oleic Acid-Coated Fe_3_O_4_ Nanoparticles. Appl. Surf. Sci. 2010, 256, 3093–3097. 10.1016/j.apsusc.2009.11.079.

[ref50] Hashemnejad S. D.; Kundu S. Rheological properties and failure of alginate hydrogels with ionic and covalent crosslinks. Soft Matter 2019, 15, 7852–7862. 10.1039/C9SM01039D. 31531488

[ref51] Basu S.; Moseson A.; Barsoum M. W. On the Determination of Spherical Nanoindentation Stress-Strain Curves. J. Mater. Res. 2006, 21, 2628–2637. 10.1557/jmr.2006.0324.

[ref52] Roa J. J.; Jiménez-Piqué E.; Capdevila X. G.; Segarra M. Nanoindentation with Spherical Tips of Single Crystals of YBCO Textured by the Bridgman Technique: Determination of Indentation Stress-Strain Curves. J. Eur. Ceram. Soc. 2010, 30, 1477–1482. 10.1016/j.jeurceramsoc.2009.10.021.

[ref53] Bakonyi I.; Péter L.; Rolik Z.; Kiss-Szabó K.; Kupay Z.; Tóth J.; Kiss L. F.; Pádár J. Decomposition of the Magnetoresistance of Multilayers into Ferromagnetic and Superparamagnetic Contributions. Phys. Rev. B 2004, 70, 054427 10.1103/PhysRevB.70.054427.

[ref54] Garcia-Torres J.; Vallés E.; Gómez E. Measurement of the Giant Magnetoresistance Effect in Cobalt–Silver Magnetic Nanostructures: Nanoparticles. Nanotechnology 2012, 23, 405701 10.1088/0957-4484/23/40/405701. 22990131

[ref55] Wang Y.-F.; Sekine T.; Takeda Y.; Yokosawa K.; Matsui H.; Kumaki D.; Shiba T.; Nishikawa T.; Tokito S. Fully Printed PEDOT:PSS-Based Temperature Sensor with High Humidity Stability for Wireless Healthcare Monitoring. Sci. Rep. 2020, 10, 2467 10.1038/s41598-020-59432-2. 32051489 PMC7016104

[ref56] Honda W.; Harada S.; Arie T.; Akita S.; Takei K. Wearable, Human-Interactive, Health-Monitoring, Wireless Devices Fabricated by Macroscale Printing Techniques. Adv. Funct. Mater. 2014, 24, 3299–3304. 10.1002/adfm.201303874.

[ref57] Tang J.; Qiao Y.; Chu Y.; Tong Z.; Zhou Y.; Zhang W.; Xie S.; Hu J.; Wang T. Magnetic Double-Network Hydrogels for Tissue Hyperthermia and Drug Release. J. Mater. Chem. B 2019, 7, 1311–1321. 10.1039/C8TB03301C. 32255170

[ref58] Müller F.; Ferreira C. A.; Azambuja D. S.; Aleman C.; Armelin E. Measuring the Proton Conductivity of Ion-Exchange Membranes Using Electrochemical Impedance Spectroscopy and Through-Plane Cell. J. Phys. Chem. B 2014, 118, 1102–1112. 10.1021/jp409675z. 24428522

