# Supplementary material for: Remote Spatiotemporal Control of a Magnetic and Electroconductive Hydrogel Network via Magnetic Fields for Soft Electronic Applications
Source: ACS Appl Mater Interfaces. 2021 Sep 1;13(36):42486–501. doi: 10.1021/acsami.1c12458 (PMC8594865; doi:10.1021/acsami.1c12458)
Supplement: Supplementary file 1 — am1c12458_si_001.pdf [file am1c12458_si_001.pdf]

SUPPORTING INFORMATION

Remote Spatio-Temporal Control of a Magnetic and Electroconductive Hydrogel Network Via Magnetic Fields for Soft Electronic Applications

Anna Puiggalí-Jou[a,◊], Ismael Babeli[a,◊], Joan Josep Roa[b,c], Justin O. Zoppe[d], Jaume Garcia-Amorós[e,f], Maria-Pau Ginebra[c,g,h], Carlos Alemán[a,c,h,*] and Jose García-Torres[c,g,*]

[a] *Departament d'Enginyeria Química, EEBE, Universitat Politècnica de Catalunya, C/ Eduard Maristany, 10-14, 08019, Barcelona, Spain*

[b] *CIEFMA (Center for Research in Structural Integrity, Reliability and Micromechanics of Materials)-Department of Materials Science and Engineering, EEBE, Universitat Politècnica de Catalunya-BarcelonaTech, 08019, Barcelona, Spain*

[c] *Barcelona Research Center in Multiscale Science and Engineering, Universitat Politècnica de Catalunya, 08930, Barcelona, Spain*

[d] *Department of Materials Science and Engineering, Universitat Politècnica de Catalunya (UPC), 08019, Barcelona, Spain*

[e] *Grup de Materials Orgànics, Departament de Química Inorgànica i Orgànica (Secció de Química Orgànica), Universitat de Barcelona, Martí i Franquès, 1, 08028, Barcelona, Spain*

[f] *Institut de Nanociència i Nanotecnologia (IN^{2}UB), Universitat de Barcelona, 08028, Barcelona, Spain*

[g] *Biomaterials, Biomechanics and Tissue Engineering Group, Department of Materials Science and Engineering, Universitat Politècnica de Catalunya (UPC), 08019, Barcelona, Spain*

[h] *Institute for Bioengineering of Catalonia (IBEC), Barcelona Institute of Science and Technology (BIST), Baldiri Reixac 10-12, 08028, Barcelona Spain*

[◊] These authors contributed equally to this work

[*] jose.manuel.garcia-torres@upc.edu and carlos.aleman@upc.edu

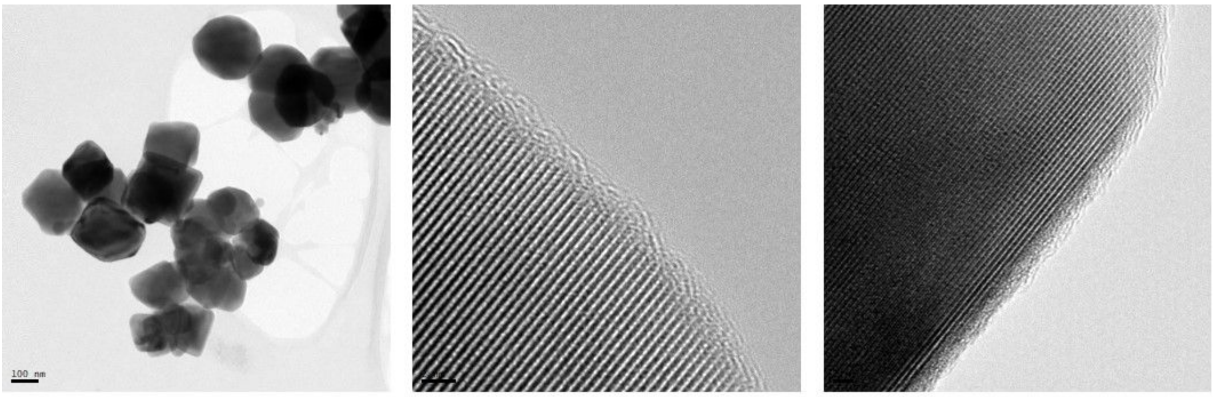

(a) **(b)** **(c)**

Figure S1. (a) TEM image of magnetite nanoparticles showing different shapes from spherical to cubic shapes. (b) and (c) HR-TEM images showing lattice fringes.

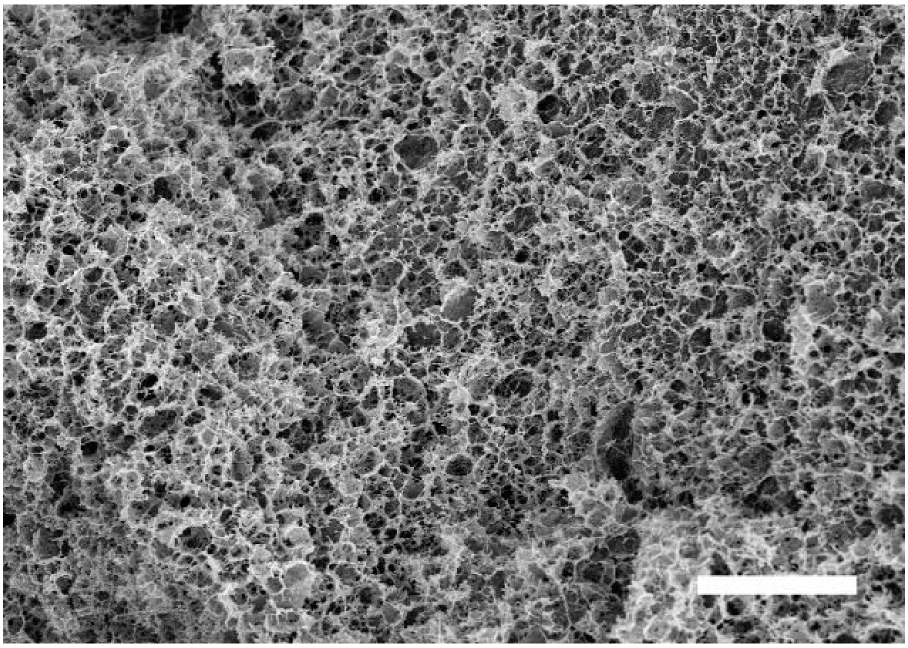

Figure S2. **S**EM image of PEDOT/Alg hydrogel without magnetite nanoparticles. Scale bar: 50 nm.

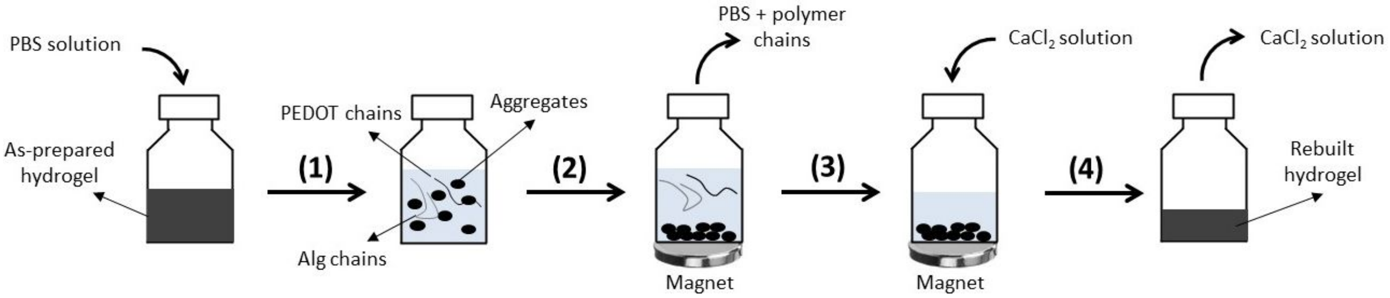

Figure S3. Scheme showing the process to disassembly and re-crosslink the hydrogel. (1) Addition of PBS solution to disassembly the hydrogel into polymer-Fe_3O_4 NPs aggregates (magnetic) as well as free Alg and PEDOT chains (non-magnetic). (2) A FeNdB magnet is put at the bottom of the vial to recover the polymer-Fe_3O_4 NPs aggregates suspended in solution since they are attracted by the magnet. The free Alg and PEDOT polymeric chains remain in suspension since they are non-magnetic. These polymeric chains are removed from the vial with PBS solution. As the magnet is present at the bottom of the vial the aggregates are attracted towards it, and therefore the final hydrogel will be more compact. (3) Addition of $CaCl_2$ solution to re-crosslink the hydrogel. The magnet is still at the bottom of the vial to avoid the dispersion of aggregates during the addition of $CaCl_2$ solution. (4) $CaCl_2$ solution is removed once the hydrogel is re-crosslinked. Note that the thickness of the rebuilt hydrogel is smaller than the as-prepared hydrogel.

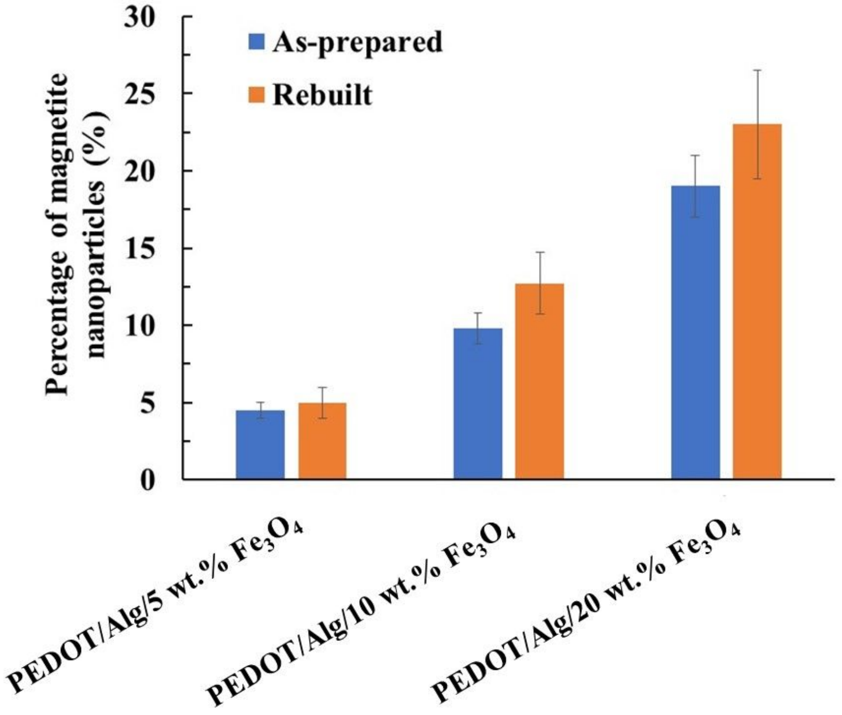

Figure S4. Graph showing the weight percentage of magnetite nanoparticles within as-prepared or rebuilt hydrogels estimated by EDX.

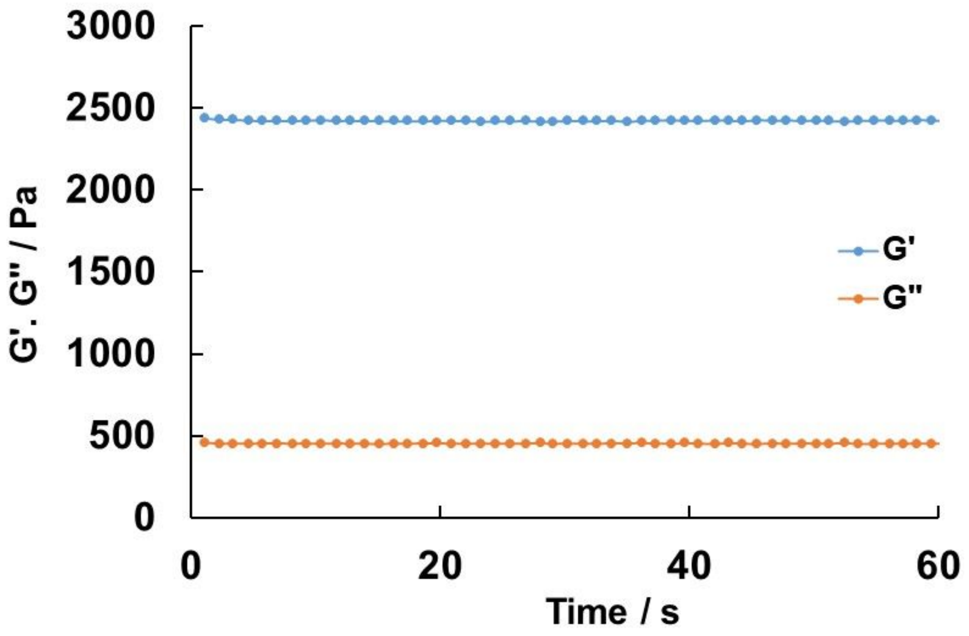

Figure S5. Evaluation of G' and G'' moduli using the oscillatory time sweep mode at a frequency of 1Hz and a displacement of $1 \cdot 10^{-3}$ rad for the as-prepared PEDOT/Alg/Fe_3O_4 NPs hydrogel.
